# Five new species of the genus *Paratemnoides* Harvey, 1991 (Pseudoscorpiones, Atemnidae) from China

**DOI:** 10.3897/BDJ.12.e124585

**Published:** 2024-06-14

**Authors:** Yanmeng Hou, Lingchen Zhao, Feng Zhang

**Affiliations:** 1 Key Laboratory of Zoological Systematics and Application, College of Life Sciences, Hebei University, Baoding, Hebei 071002, P. R. China, Baoding, China Key Laboratory of Zoological Systematics and Application, College of Life Sciences, Hebei University, Baoding, Hebei 071002, P. R. China Baoding China; 2 College of Life Sciences, Capital Normal University, 105 Xisanhuanbeilu, Haidian District, Beijing, China College of Life Sciences, Capital Normal University, 105 Xisanhuanbeilu, Haidian District Beijing China; 3 Changzhi Tunliu District No. 6 Middle School, Changzhi, China Changzhi Tunliu District No. 6 Middle School Changzhi China

**Keywords:** morphology, new species, pseudoscorpion, taxonomy

## Abstract

**Background:**

*Paratemnoides* Harvey, 1991 is currently represented by 28 species and two subspecies, which are widespread in the world, except for Europe and Antarctica. *Paratemnoidessinensis* (Beier, 1932) represents the only species of this genus currently recorded from China.

**New information:**

Five new *Paratemnoides* species collected from China are described, including detailed diagnoses and illustrations: *P.guangdongensis* sp. nov. from Guangdong, *P.parvus* sp. nov., *P.politus* sp. nov. and *P.yunnanensis* sp. nov. from Yunnan and *P.trisulcus* sp. nov. from Guangxi. An identification key to all known *Paratemnoides* species from China and a distribution map are also provided.

## Introduction

The pseudoscorpion family Atemnidae Kishida, 1929, belonging to the superfamily Cheliferoidea Risso, 1827, is currently represented by two subfamilies (Atemninae Beier, 1932 and Miratemninae Beier, 1932). The reciprocal monophyly of the two subfamilies remains to be confirmed and it is generally assumed that the main difference between them is the position of the tactile setae on the tarsus of leg Ⅳ (near base vs. near middle) and the number of setae on the cheliceral hand (four vs. five) (Kishida 1929, Chamberlin 1933, Dumitresco and Orghidan 1970, Harvey 1991, Harvey 1992, Klausen 2005). Atemnidae can be easily distinguished from the other three families in Cheliferoidea by the position of venom apparatus: venom apparatus only present in fixed chelal finger vs. only present in movable chelal finger (Chernetidae Menge, 1855) or in both chelal fingers (Cheliferidae Risso, 1827 and Withiidae Chamberlin, 1931). At present, Atemnidae contains 20 extant genera and one fossil genus (*Progonatemnus* Beier, 1955), with a total of 187 species (15 species in five genera are known from China). Of these, 15 genera and 165 species belong to the subfamily Atemninae and the rest belong to the subfamily Miratemninae (WPC 2024).

*Paratemnoides* Harvey, 1991, one of the genera within the subfamily Atemninae, is currently represented by 28 species, which are widespread in the world, except for Europe and Antarctica. The main distribution of countries belonging to Asia are Cambodia, India, Indonesia, Japan, Laos, Malaysia, Myanmar, Philippines, Singapore and Thailand. *Paratemnoidessinensis* (Beier, 1932) represents the only species of this genus currently distributed in China (WPC 2024). The genus can be diagnosed as follows: carapace smooth and glossy, without furrow; tergites incompletely divided; pedipalp stout, surface smooth or with granulations on prolateral surface; palpal trochanter with two well-developed granular tubercles; trichobothrium *it* situated near the middle of the fixed chelal finger and distance between *it* and fingertip further than distance between *ist* and *isb*; *st* closer to *sb* than to *t*; legs moderately stout, a long tactile seta of leg IV situated near the base of tarsal segment (Beier 1932, Murthy and Ananthakrishnan 1977, Mathew and Joseph 2019).

During the identification of atemnid pseudoscorpion specimens collected in 2018 and 2019, five new species, belonging to *Paratemnoides* were found and which are described with detailed diagnoses, descriptions and illustrations.

## Materials and methods

The specimens were preserved in 75% ethanol and deposited in the Museum of Hebei University (MHBU) (Baoding, China). Photographs were taken with a Leica M205A stereomicroscope equipped with a Leica DFC550 camera and the LAS software v. 4.6 and the Leica M205A stereomicroscope with a drawing tube was used for drawings and measurements. The chela and the chelal hand were measured in ventral view. Detailed examination was carried out with an Olympus BX53 compound light microscope. Temporary slide mounts were made in glycerol.

Terminology and measurements mostly follow Chamberlin (1931), with some minor modifications to the terminology of trichobothria (Harvey 1992), chelicerae (Judson 2007) and male genitalia (Klausen 2005). All measurements are given in mm.

The following abbreviations are used for the trichobothria: **b**, basal; **sb**, sub-basal; **st**, sub-terminal; **t**, terminal; **ib**, interior basal; **isb**, interior sub-basal; **ist**, interior sub-terminal; **it**, interior terminal; **eb**, exterior basal; **esb**, exterior sub-basal; **est**, exterior sub-terminal; **et**, exterior terminal. Cheliceral setae: **es**, exterior seta; **is**, interior seta; **ls**, laminal seta; **bs**, basal seta; **sbs**, sub-basal seta. Male genitalia: **a**, lateral apodeme; **br**, hooked branch; **c**, sclerotised bar; **d**, longitudinal fold of medial diverticulum; **e**, ejaculatory canal atrium; **f**, lateral rods; **g**, dorsal apodeme; **h**, ventral diverticulum; **l**, lateral lip of lateral apodeme.

## Taxon treatments

### Paratemnoides guangdongensis

Hou, Zhao & Zhang sp. nov.

963224A7-4565-58EE-8510-12CE3C49C084

920EA9F0-4ABF-4BAE-BD48-F634EB5BCEDC

#### Materials

- **Type status:** Holotype. **Occurrence:** recordedBy: Xiangbo Guo; individualCount: 1; sex: male; lifeStage: adult; occurrenceID: 22FEED8C-8AF0-55E7-886A-9F2C6B9CA28B; **Taxon:** scientificName: *Paratemnoidesguangdongensis*; **Location:** country: China; stateProvince: Guangdong; locality: Meijiang District, Pankeng Park; verbatimElevation: 232 m; verbatimCoordinates: 24°14.474′N, 116°8.377′E; **Event:** year: 2018; month: 4; day: 10; **Record Level:** institutionID: the Museum of Hebei University (MHBU); institutionCode: MHBU-GD180410-0201
- **Type status:** Paratype. **Occurrence:** recordedBy: Xiangbo Guo; individualCount: 13; sex: 13 females; lifeStage: adult; occurrenceID: FBD42E2F-F094-504B-B705-B0EEA49524D9; **Taxon:** scientificName: *Paratemnoidesguangdongensis*; **Location:** country: China; stateProvince: Guangdong; locality: Meijiang District, Pankeng Park; verbatimElevation: 232 m; verbatimCoordinates: 24°14.474′N, 116°8.377′E; **Event:** year: 2018; month: 4; day: 10; **Record Level:** institutionID: the Museum of Hebei University (MHBU); institutionCode: MHBU-GD180410-020102–14

#### Description

**Figure 1.**
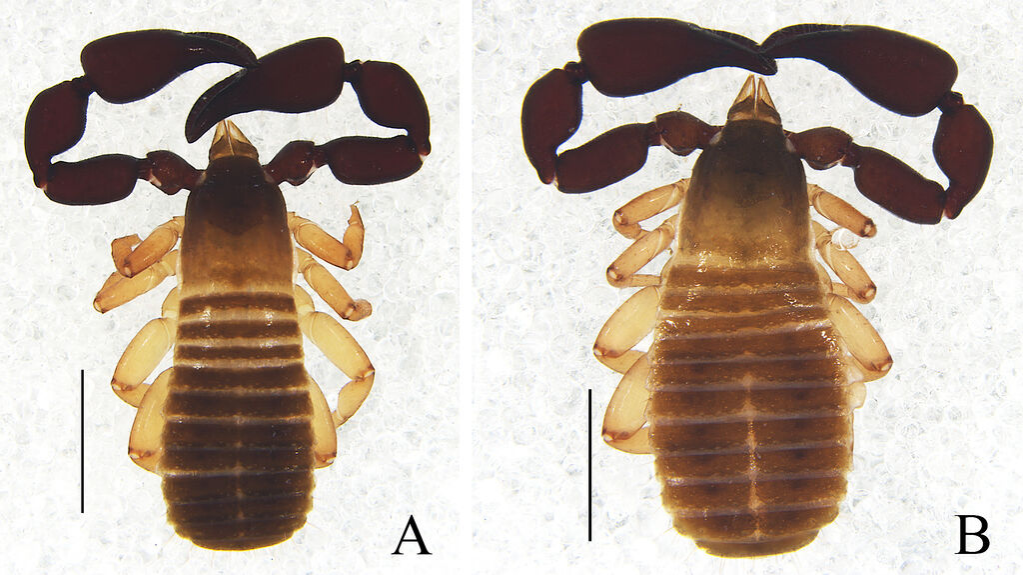
*Paratemnoidesguangdongensis* sp. nov. **A** holotype male, habitus (dorsal view); **B** paratype female, habitus (dorsal view). Scale bars: 1.00 mm.

**Figure 2.**
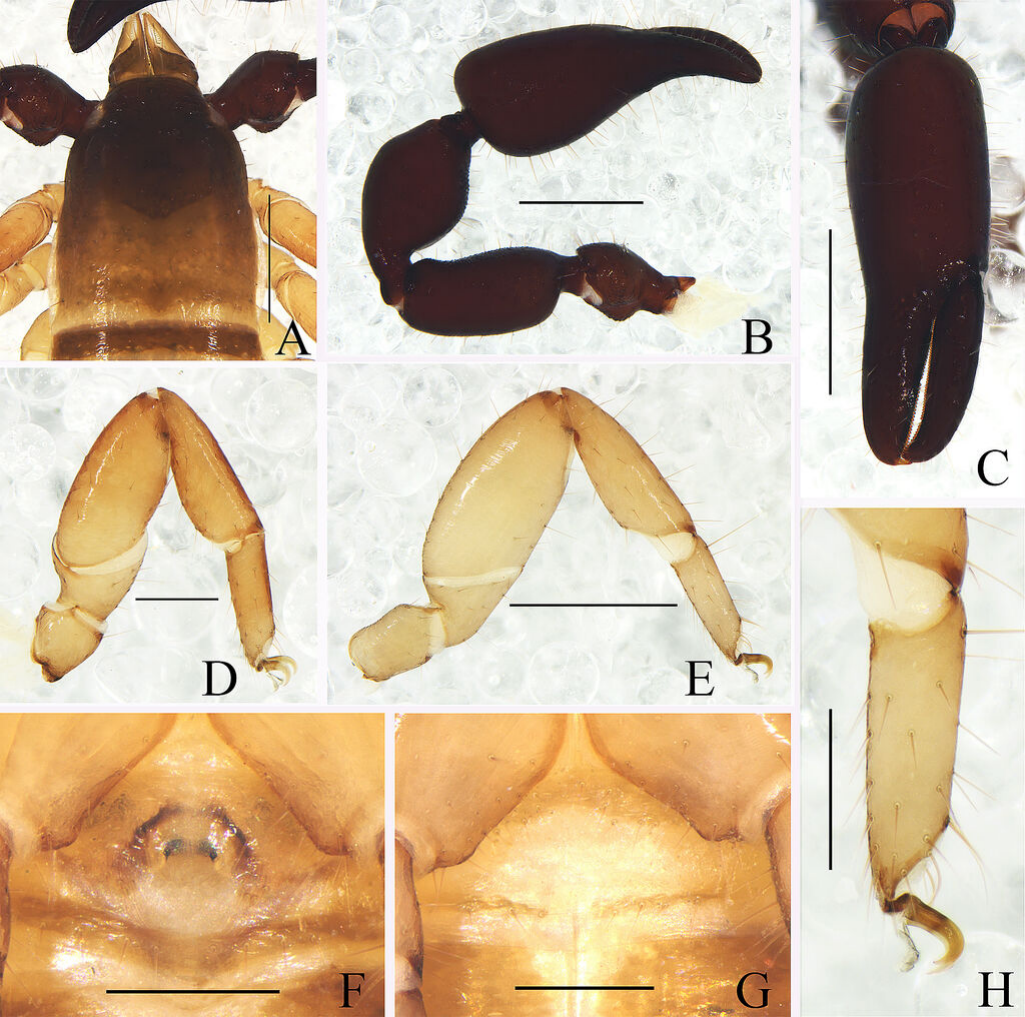
*Paratemnoidesguangdongensis* sp. nov., holotype male (**A–F, H**), paratype female (**G**) **A** carapace (dorsal view); **B** left pedipalp (dorsal view); **C** left chela (lateral view); **D** left leg I (lateral view); **E** left leg IV (lateral view); **F** male genital area (ventral view); **G** female genital area (ventral view); **H** tarsus of left leg IV (lateral view). Scale bars: 0.50 mm (**A–C, E**); 0.20 mm (**D, F–H**).

**Figure 3.**
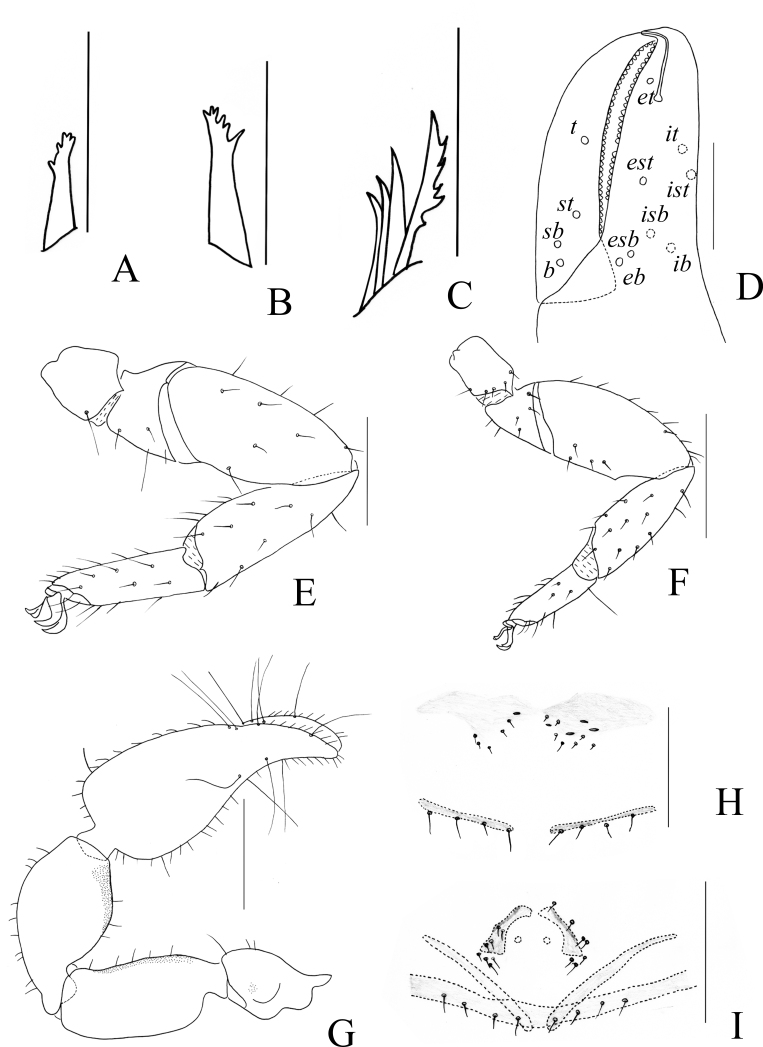
*Paratemnoidesguangdongensis* sp. nov., holotype male (**A, C–G, I**), paratype female (**B, H**) **A** male galea; **B** female galea; **C** rallum; **D** left chelal fingers (lateral view), with details of trichobothrial pattern; **E** left leg I (lateral view); **F** left leg IV (lateral view); **G** left pedipalp (dorsal view); **H** female genital area (ventral view); **I** male genital area (ventral view). Scale bars: 0.50 mm (**F, G**); 0.25 mm (**D, E, H, I**); 0.10 mm (**A–C**).

Male (holotype) (Fig. 1A, Fig. 2A–F, H, Fig. 3A, C–G and I). Colour: anterior half of carapace, tergites and palpal coxa dark brown, but paler in posterior half of carapace; pedipalps dark reddish-brown; chelicerae light brown; legs and pleural membrane light yellow.

Carapace (Fig. 2A): 1.19× longer than broad; surface smooth, without furrow; anterior half darker than posterior half, dividing line M-shape; with two distinct eyespots situated near anterior margin of carapace; anterior margin with six setae, posterior margin with nine setae, 63 in total, each seta acicular and very slightly curved.

Chelicera (Fig. 3A and C): surface smooth; four setae (*sbs* absent) and two lyrifissures (exterior condylar lyrifissure and exterior lyrifissure) present on hand; movable finger with one galeal seta (short and acute); *bs* and *es* short and dentate apically, *is* and *ls* long and acute; galea present, shorter and with six short branchlets (Fig. 3A). Serrula interior connected to fixed finger for entire length, proximally modified to form velum, serrula exterior with 21 blades, the basal one longest; lamina exterior present. Rallum composed of four blades, the basal two blades shorter than others, the distal one dentated anteriorly, remainder smooth (Fig. 3C).

Pedipalp (Fig. 2B–C, Fig. 3D and G): stout, trochanter 1.65×, femur 2.20×, patella 1.81×, chela with pedicel (without pedicel) 2.73× (2.47×), hand with pedicel (without pedicel) 1.65× (1.39×) longer than broad; movable chelal finger 0.69× (0.82×) longer than hand with pedicel (without pedicel) and 0.42× (0.46×) longer than chela with pedicel (without pedicel). Setae generally long and acuminate. Retrolateral surface of trochanter, prolateral surface of femur and patella granular; trochanter with two well-developed conical tubercles; middle part of patella intumescent and spherical. Fixed chelal finger with eight trichobothria, movable chelal finger with four trichobothria: *eb* and *esb* situated at base of fixed finger on retrolateral face, *esb* slightly distal to *eb*; *ib* and *isb* situated at base of fixed finger on prolateral face, *isb* slightly distal to *ib*; *est* in the middle of fixed finger; *et* near sub-distal of fixed finger; *est* closer to *esb* than to *et*; *it* distal to *est* and proximal to *et*; *ist* proximal to *it* and slightly distal to *est*; *it* closer to *ist* than to fingertip; distance between *est* and *esb* nearly equal to that of *ist* and *isb*; distance between *it* and fingertip further than distance between *ist* and *isb*; *b* and *sb* situated at base of movable finger on retrolateral face; *t* in the middle of movable finger and at same level as *it*; *sb* closer to *b* than to *st*; *st* closer to *sb* than to *t* (Fig. 3D). Venom apparatus only present in fixed chelal finger, venom ducts curved and short, terminating in inflated nodus ramosus between *et* and *est*, closer to *et.* Both chelal fingers with a row of acute teeth, spaced contiguously along the margin, slightly rounded proximally: fixed chelal finger with 38–39 teeth; movable chelal finger with 54–56 teeth (nearly as large as teeth on fixed chelal finger); without accessory teeth (Fig. 3D). Femur without long tactile setae. Movable chelal finger slightly curved in lateral view (Fig. 3D).

Opisthosoma: generally typical, all setae long and acuminate; pleural membrane longitudinally striate, without setae. Tergites I–V and XI undivided and others incompletely divided, setal bases distinct larger, tergal chaetotaxy I–XI: 9: 10: 10: 14: 14: 12: 16: 13: 14: 16 (4T): 14 (2T). All sternites (except sternite XI) divided, darker and darker from sternites Ⅳ to sternites XI, each half sternite with seven long setae, sternites X and XI each with four tactile setae. Anus (tergite Ⅻ and sternite Ⅻ) without raised rim. Anterior genital operculum with four setae on each side, posterior margin with six setae, arranged in a row.

Legs (Fig. 2D–E, H and Fig. 3E–F): generally typical, fairly smooth, slightly stout; leg I darker than leg Ⅳ; junction between femora and patellae I and II oblique. Femoropatella of leg Ⅳ 2.56× longer than deep; tibia 2.95× longer than deep; with basal tactile setae on tarsal segment: tarsus 3.25× longer than deep (TS = 0.13); subterminal tarsal setae arcuate and acute. Arolium slightly shorter than claws, not divided; claws smooth.

Dimensions (length/breadth or, in the case of the legs, length/depth in mm). Male (females in parentheses): body length 2.77 (2.90–3.36). Carapace 0.99/0.83 (1.00–1.02/0.96–0.99). Pedipalp: trochanter 0.51/0.31 (0.47–0.49/0.30–0.31), femur 0.77/0.35 (0.76–0.78/0.34–0.37), patella 0.78/0.43 (0.76–0.79/0.41–0.44), chela (with pedicel) 1.34/0.49 (1.42–1.48/0.50–0.57), chela (without pedicel) 1.21 (1.28–1.34), hand (with pedicel) 0.81 (0.95–1.02), movable finger length 0.56 (0.57–0.59). Leg I: trochanter 0.18/0.17 (0.18–0.19/0.15–0.18), femur 0.28/0.23 (0.27–0.28/0.21–0.23), patella 0.47/0.21 (0.45–0.47/0.20–0.21), tibia 0.43/0.15 (0.42/0.14), tarsus 0.33/0.10 (0.31–0.34/0.09–0.10). Leg IV: trochanter 0.30/0.19 (0.33/0.21–0.22), femoropatella 0.82/0.32 (0.88–0.90/0.34–0.36), tibia 0.59/0.20 (0.63/0.20), tarsus 0.39/0.12 (0.39–0.41/0.12).

#### Diagnosis

This new species is characterised by (see taxon discussion for more details): anterior half of carapace darker than posterior half, with two distinct eyespots; pedipalp stout, palpal femur 2.20 (♂), 2.11–2.24 (♀), chela with pedicel 2.73 (♂), 2.60–2.84 (♀), chela without pedicel 2.47 (♂), 2.35–2.56 (♀) × longer than broad; dorsal tubercle on trochanter well-developed; movable chelal finger with 54–56 teeth; retrolateral surface of trochanter, prolateral surface of patella, femur and hand granular; middle part of patella intumescent and spherical.

#### Etymology

Named after the type locality, Guangdong (China).

#### Distribution

China (Guangdong) (Fig. 16).

#### Taxon discussion

Before this study, a total of 28 *Paratemnoides* species have been recorded around the world, of which 16 and one subspecies come from Asia (only one species, *P.sinensis*, comes from China). *Paratemnoidesguangdongensis* sp. nov. is similar to *P.parvus* sp. nov., but differs by slender chela (♂) (e.g. chela with pedicel 2.73× vs. 2.54–2.66× longer than broad) and more movable chelal finger teeth (♂) (54–56 vs. 41–42).

*Paratemnoidesguangdongensis* sp. nov. can be distinguished from *P.assimilis* (Beier, 1932) by the number of posterior margin setae on the carapace (9 vs. 6), slightly smaller body size and stouter pedipalps (♂) (e.g. body length 2.77 mm vs. 3.30 mm; chela with pedicel 2.73× vs. 2.60× longer than broad; chelal hand with pedicel 1.65× vs. 1.88× longer than broad, length 0.81 mm vs. 0.94 mm); from *P.borneoensis* (Beier, 1932) by the number of serrula exterior blades (21 vs. 24), smaller body size and slender pedipalps (♀) (e.g. body length 2.90–3.36 mm vs. 3.50 mm; palpal femur length 0.76–0.78 mm vs. 0.68 mm); from *P.curtulus* (Redikorzev, 1938) by the arrangement of trichobothria (e.g. distance between *est* and *esb* nearly equal to that of *ist* and *isb* vs. shorter to that of *ist* and *isb*) and the slender chela (♂) (chela with pedicel 2.73× vs. 2.19× longer than broad, length 1.34 mm vs. 1.05 mm); from *P.indicus* (Sivaraman, 1980) by the presence of more setae on the carapace (63 vs. 46), more movable chelal finger teeth (54–56 vs. 42) and smaller body size (e.g. body length (♂) 2.77 mm vs. 4.02 mm, (♀) 2.90–3.36 mm vs. 3.50 mm; palpal femur (♀) 2.11–2.24× vs. 2.00× longer than broad); from *P.japonicus* (Morikawa, 1953) by the trait of eyes (with two distinct eyespots vs. eyespots absent), the number of serrula exterior blades (21 vs. 18) and slightly smaller body size (♂) (e.g. body length 2.77 mm vs. 2.97 mm; palpal femur 2.20× vs. 2.40× longer than broad, length 0.77 mm vs. 0.81 mm); from *P.laosanus* (Beier, 1951) by the presence of more chelal fingers teeth (fixed chelal finger with 38–39 vs. 33 teeth; movable chelal finger with 54–56 vs. 44 teeth) and smaller body length (♂♀) (2.77–3.36 mm vs. 3.50–4.00 mm); from *P.mahnerti* (Sivaraman, 1981) by the presence of more setae on the carapace (63 vs. 40), the number of serrula exterior blades (21 vs. 19) and relative position of trichobothrium *st* (*st* situated closer to *sb* than to *t* vs. midway between *sb* and *t*); from *P.pallidus* (Balzan, 1892) by the number of serrula exterior blades (21 vs. 25), the presence of more movable chelal finger teeth (54–56 vs. 45 teeth) and smaller body size (♀) (e.g. body length 2.90–3.36 mm vs. 4.30 mm); from *P.philippinus* (Beier, 1932) by the number of serrula exterior blades (21 vs. 26) and smaller body size (♂) (e.g. body length 2.77 mm vs. 3.50 mm; chela with pedicel 2.73× vs. 2.20× longer than broad; chelal hand length 0.81 mm vs. 0.90 mm; movable chelal finger length 0.56 mm vs. 0.60 mm); from *P.plebejus* (With, 1906) by darker body colour (carapace and tergites dark brown vs. yellowish brown), smaller body size (♀) (e.g. body length 2.90–3.36 mm vs. 4.00–5.70 mm; palpal femur 2.11–2.24× vs. 2.00× longer than broad), more chelal fingers teeth (♂) (fixed finger with 38–39 vs. 32 teeth; movable chelal finger with 54–56 vs. 46 teeth) and the trait of pedipalp (prolateral surface of femur and patella granular only vs. almost entire surface granular); from *P.pococki* (With, 1907) by darker body colour (carapace and tergites dark brown vs. pale brown) and smaller body size and slender pedipalps (♀) (body length 2.90–3.36 mm vs. 3.87 mm; palpal femur length 0.76–0.78 mm vs. 0.62 mm; movable chelal finger length 0.57–0.59 mm vs. 0.46 mm); from *P.politus* sp. nov. by slender pedipalps (♂) (e.g. palpal femur 2.20× vs. 1.94–2.00× longer than broad) and more movable chelal finger teeth (54–56 vs. 48–50); from *P.redikorzevi* (Beier, 1951) by more chelal fingers teeth (fixed finger with 38–39 vs. 35 teeth; movable chelal finger with 54–56 vs. 48 teeth), slightly smaller body size and slender pedipalps (e.g. body length (♂) 2.77 mm vs. 3.00 mm; palpal femur (♀) length 0.76–0.78 mm vs. 0.70–0.72 mm; movable finger length 0.57–0.59 mm vs. 0.51–0.57 mm); from *P.robustus* (Beier, 1932) by the number of serrula exterior blades (21 vs. 26) and smaller body length (e.g. body length (♀) 2.90–3.36 mm vs. 4.60 mm; palpal femur (♂) 2.20× vs. 2.28× longer than broad, length 0.77mm vs. 0.91 mm; movable finger length 0.56 mm vs. 0.66 mm); from *P.salomonis* (Beier, 1935) by the trait of eyespots (with two distinct eyespots vs. eyespots wanting), smaller body length and the slender pedipalps (♂) (e.g., body length 2.77 mm vs. 3.50 mm; palpal femur 2.20× vs. 2.10× longer than broad, length 0.77 mm vs. 0.69 mm; chela with pedicel 2.73× vs. 2.60× longer than broad; chela without pedicel 2.47× vs. 2.40× longer than broad); from *P.sinensis* by the presence of more movable chelal finger teeth (54–56 vs. 43) ; from *P.sumatranus* (Beier, 1935) by the number of serrula exterior blades (21 vs. 24) and smaller body size (♂) (e.g. body length 2.77 mm vs. 2.50 mm; palpal femur 2.20× vs. 2.30× longer than broad, length 0.77 mm vs. 0.61 mm; movable chelal finger length 0.56 mm vs. 0.47 mm); from *P.trisulcus* sp. nov. by stouter pedipalps (♂) (e.g. palpal femur 2.20× vs. 2.39× longer than broad); from *P.yunnanensis* sp. nov. by slender chela (♂) (e.g. chela with pedicel 2.73× vs. 2.63–2.64× longer than broad) (With 1906, With 1907, Beier 1932, Beier 1935a, Beier 1935b, Redikorzev 1938, Beier 1951, Morikawa 1953, Sivaraman 1980, Sivaraman 1981, Mathew and Joseph 2019).

### Paratemnoides parvus

Hou, Zhao & Zhang sp. nov.

1C28506F-3263-5E4E-8C2F-22869F72439B

A3AE6C86-BC3B-409E-8020-89E9CDAF3727

#### Materials

- **Type status:** Holotype. **Occurrence:** recordedBy: Yannan Mu; individualCount: 1; sex: male; lifeStage: adult; occurrenceID: 3248D9E3-FADC-5225-B70E-2722132E534A; **Taxon:** scientificName: *Paratemnoidesparvus*; **Location:** country: China; stateProvince: Yunnan; county: Mengla; locality: Menglun Town, Xishuangbanna Tropical Botanical Garden; verbatimElevation: 567 m; verbatimCoordinates: 21°55.416′N, 101°16.097′E; **Event:** year: 2019; month: 8; day: 13; **Record Level:** institutionID: the Museum of Hebei University (MHBU); institutionCode: MHBU-YNML19081301
- **Type status:** Paratype. **Occurrence:** recordedBy: Yannan Mu; individualCount: 2; sex: 2 males; lifeStage: adult; occurrenceID: 46B7C7EA-9482-5E8B-9205-063B681D382A; **Taxon:** scientificName: *Paratemnoidesparvus*; **Location:** country: China; stateProvince: Yunnan; county: Mengla; locality: Menglun Town, Xishuangbanna Tropical Botanical Garden; verbatimElevation: 567 m; verbatimCoordinates: 21°55.416′N, 101°16.097′E; **Event:** year: 2019; month: 8; day: 13; **Record Level:** institutionID: the Museum of Hebei University (MHBU); institutionCode: MHBU-YNML19081347–48
- **Type status:** Paratype. **Occurrence:** recordedBy: Yannan Mu; individualCount: 12; sex: 12 females; lifeStage: adult; occurrenceID: 0A6DF39C-89A3-50AA-8797-C923EB352E00; **Taxon:** scientificName: *Paratemnoidesparvus*; **Location:** country: China; stateProvince: Yunnan; county: Mengla; locality: Menglun Town, Xishuangbanna Tropical Botanical Garden; verbatimElevation: 567 m; verbatimCoordinates: 21°55.416′N, 101°16.097′E; **Event:** year: 2019; month: 8; day: 13; **Record Level:** institutionID: the Museum of Hebei University (MHBU); institutionCode: MHBU-YNML19081349–60

#### Description

**Figure 4.**
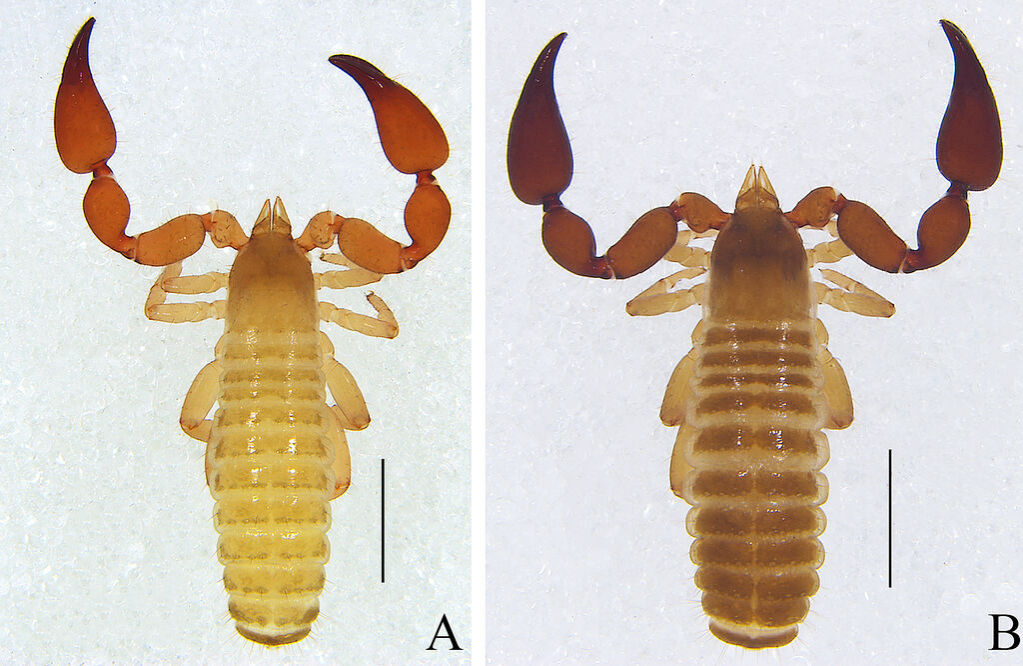
*Paratemnoidesparvus* sp. nov. **A** holotype male, habitus (dorsal view); **B** paratype female, habitus (dorsal view). Scale bars: 1.00 mm.

**Figure 5.**
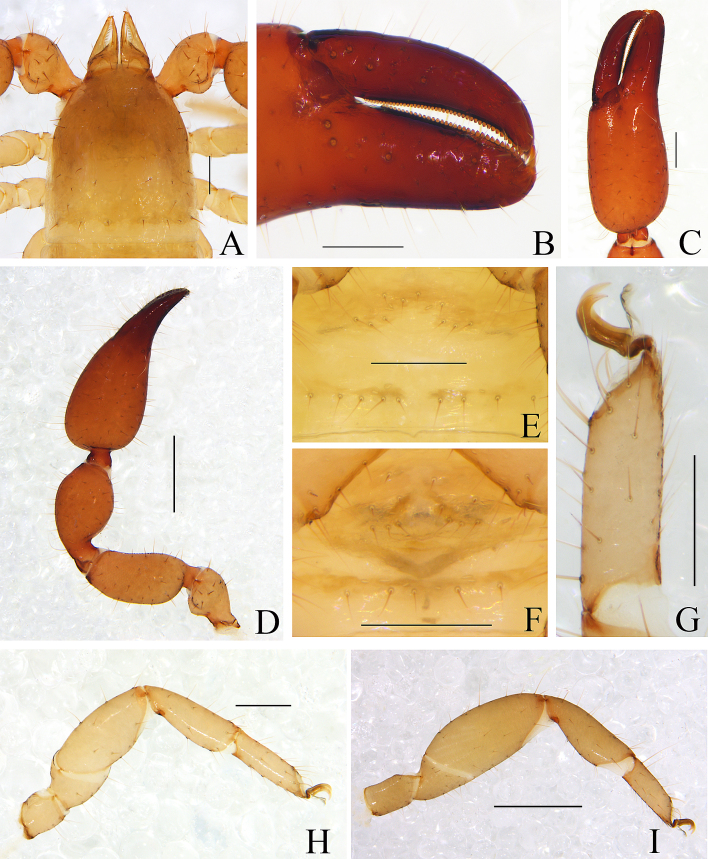
*Paratemnoidesparvus* sp. nov., holotype male (**A–D, F–I**), paratype female (**E**) **A** carapace (dorsal view); **B** left chelal fingers (lateral view); **C** left chela (lateral view); **D** left pedipalp (dorsal view); **E** female genital area (ventral view); **F** male genital area (ventral view); **G** tarsus of left leg IV (lateral view); **H** left leg I (lateral view); **I** left leg IV (lateral view). Scale bars: 0.50 mm (**D, I**); 0.20 mm (**A–C, E–H**).

**Figure 6.**
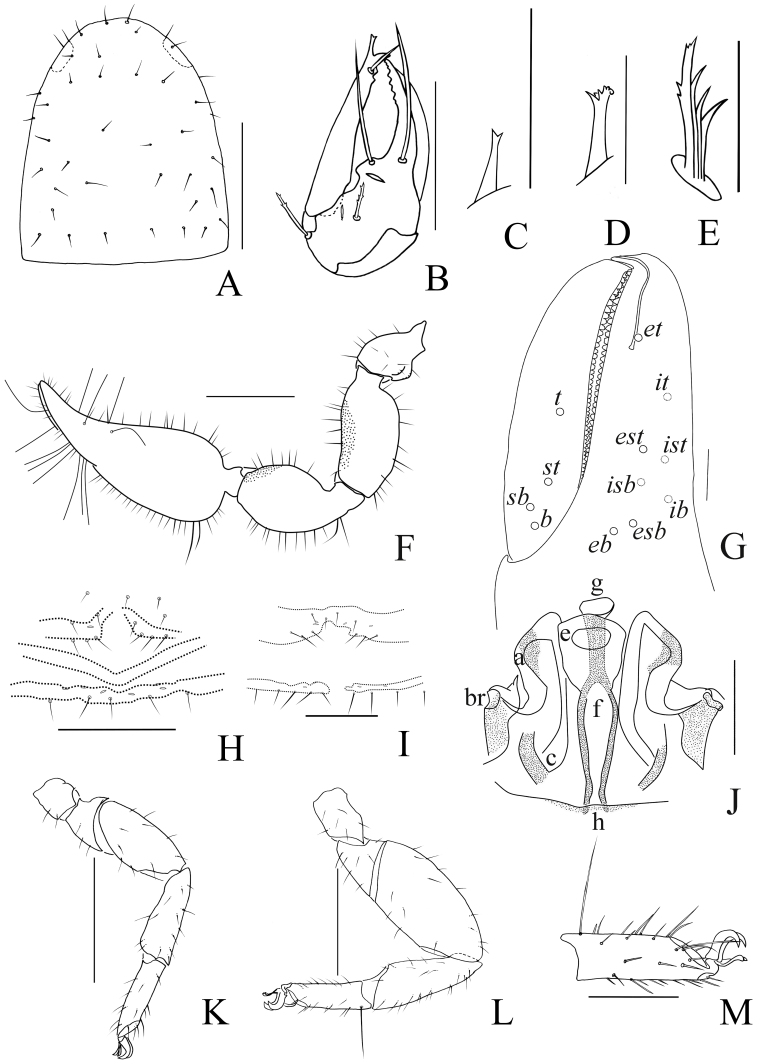
*Paratemnoidesparvus* sp. nov., holotype male (**A–C, E–H, J–M**), paratype female (**D, I**) **A** carapace (dorsal view); **B** left chelicera (dorsal view); **C** male galea; **D** female galea; **E** rallum; **F** left pedipalp (dorsal view); **G** left chelal fingers (lateral view), with details of trichobothrial pattern; **H** male genital area (ventral view); **I** female genital area (ventral view); **J** male genital organ; **K** left leg I (lateral view); **L** left leg IV (lateral view); **M** tarsus of left leg IV (lateral view). Scale bars: 1 mm (**F**); 0.50 mm (**A**); 0.25 mm (**B, G, J**); 0.20 mm (**H, I, K–M**); 0.10 mm (**C–E**).

Males (holotype and paratypes) (Fig. 4A, Fig. 5A–D, F–I, Fig. 6A–C, E–H and J–M). Colour: pedipalps reddish-brown, remainder yellowish-brown, but paler in posterior half of carapace.

Carapace (Figs 5, 6A): 1.13–1.17× longer than broad; surface smooth, without furrow; anterior half darker than posterior half; with two distinct eyespots situated near anterior margin of carapace; anterior margin with four setae, posterior margin with seven or eight setae, 41–42 in total, each seta acicular and very slightly curved.

Chelicera (Fig. 6B–C and E): much smaller than carapace length; surface smooth; four setae (*sbs* absent; *bs* shorter than others) and two lyrifissures (exterior condylar lyrifissure and exterior lyrifissure) present on hand; movable finger with one slightly curved galeal seta; *bs* and *es* dentate apically, *is* and *ls* long and acute. Fixed finger with four or five large retrorse teeth and three small apical teeth, movable finger with a long broadly dentated subapical lobe and two or three small sub-terminal teeth; galea present, shorter and with two small terminal branchlets and two small lateral dentations (Fig. 6C). Serrula interior connected to fixed finger for entire length, proximally modified to form velum, serrula exterior with 22–26 blades, the basal one longest; lamina exterior present. Rallum composed of four blades, the basal two blades shorter than others, the distal one dentated anteriorly, remainder smooth (Fig. 6E).

Pedipalp (Fig. 5B–D and Fig. 6F–G): stout, trochanter 1.34–1.39×, femur 2.11–2.36×, patella 1.74–1.84×, chela with pedicel (without pedicel) 2.54–2.66× (2.43–2.52×), hand with pedicel (without pedicel) 1.66–1.68× (1.46–1.47×) longer than broad; movable chelal finger 0.65–0.71× (0.74–0.82×) longer than hand with pedicel (without pedicel) and 0.40–0.45× (0.43–0.48×) longer than chela with pedicel (without pedicel). Setae generally long and acuminate. Only prolateral surface of femur and patella granular; trochanter with two well-developed conical tubercles. Fixed chelal finger with eight trichobothria, movable chelal finger with four trichobothria: *eb* and *esb* situated at base of fixed finger on retrolateral face, *esb* slightly distal to *eb*; *ib* and *isb* situated at base of fixed finger on prolateral face, *isb* slightly distal to *ib*; *est* in the middle of fixed finger; *et* near sub-distal of fixed finger; *est* closer to *esb* than to *et*; *it* distal to *est* and proximal to *et*; *ist* slightly proximal to *est*; *it* closer to *ist* than to fingertip; distance between *est* and *esb* further from that of *ist* and *isb*; distance between *it* and fingertip further than distance between *ist* and *isb*; *b* and *sb* situated at base of movable finger on retrolateral face; *t* in the middle of movable finger and at same level as *it*; *sb* closer to *b* than to *st*; *st* closer to *sb* than to *t* (Fig. 6G). Venom apparatus only present in fixed chelal finger, venom ducts curved and short, terminating in inflated nodus ramosus between *et* and *est*, closer to *et.* Both chelal fingers with a row of acute teeth, spaced contiguously along the margin, slightly rounded proximally: fixed chelal finger with 32–34 teeth; movable chelal finger with 41–42 teeth (nearly as large as teeth on fixed chelal finger); without accessory teeth (Fig. 6G). Femur without long tactile setae. Movable chelal finger slightly curved in lateral view (Fig. 5B and Fig. 6G).

Opisthosoma: generally typical, all setae long, acuminate and biseriate; pleural membrane longitudinally striate, without setae. Tergites I–Ⅱ and XI undivided and others incompletely divided, tergal chaetotaxy I–XI: 8: 8: 3–4: 5–5: 6–7: 5–7: 5–6: 5–6: 5–6: 5–6 + (4T): 13(2T). All sternites (except sternite XI) divided, sternal chaetotaxy IV–XI: 4–4: 7–8: 5–8: 5–7: 6–8: 6–8: 4–6 (4T): 11 (4T). Anus (tergite Ⅻ and sternite Ⅻ) without raised rim. Anterior genital operculum with eight or nine setae on each side, posterior margin with seven setae. Male genitalia (Fig. 5F, Fig. 6H and J): lateral apodemes (a) relatively small; the hooked branch (br) well-developed, bowed distally and terminated in a plate-like tip; the proximal part with a nearly pale sclerotised bar (c), distinctly curved; the longitudinal fold of medial diverticula (d) vestigial; the ejaculatory canal atrium (e) not well-developed, curved distally; the lateral rods (f) short and diverging proximally; the tip of dorsal apodeme (g) completely joined; the ventral diverticulum (h) bilobed; genital atrium without genital setae.

Legs (Fig. 5G–I and Fig. 6K–M): generally typical, fairly smooth, slightly stout; junction between femora and patellae I and II oblique. Femoropatella of leg Ⅳ 2.68–2.69× longer than deep; tibia 3.00–3.06× longer than deep; with basal tactile setae on tarsal segment: tarsus 3.17–3.25× longer than deep (TS = 0.15–0.16); subterminal tarsal setae arcuate and acute. Arolium slightly shorter than claws, not divided; claws smooth.

Adult females (Fig. 4B, Fig. 5E and Fig. 6I): Mostly same as the males, but a little smaller and darker. Chelicera: hand with four setae; galea with six branchlets; serrula exterior with 22–23 blades. Pedipalps: stout, trochanter 1.54, femur 2.23–2.29, patella 1.87–1.90, chela (with pedicel) 2.71–2.75, chela (without pedicel) 2.55–2.57, hand (without pedicel) 1.51× longer than broad, movable chelal finger 0.71–0.75× longer than hand without pedicel; dorsal tubercle on trochanter not as well developed as that of males. Opisthosoma: tergites I–Ⅲ and XI undivided and others incompletely divided, tergal chaetotaxy I–XI: 8: 8: 9: 10–12: 12–13: 6–8: 6–8: 6–8: 6–7: 5–7 (4T): 10–11 (2T). All sternites (except sternite XI) divided, sternal chaetotaxy IV–XI: 4–5: 8–9: 6–9: 6–7: 5–9: 6–8: 5–6 (4T): 10–11 (4T). Female genitalia: simple, spermathecae provided with separated median cribriform plates; anterior genital operculum with ten setae, posterior margin with nine setae; with two or three lyrifissures on both operculums. Legs: femoropatella of leg Ⅳ 2.68× longer than deep; tibia 2.95–3.00× longer than deep; with basal tactile setae on tarsal segment: tarsus 3.23× longer than deep.

Dimensions (length/breadth or, in the case of the legs, length/depth in mm; ratios in parentheses). Males (females in parentheses): body length 3.39–3.49 (3.17–3.26). Carapace 0.81/0.72–0.69 (0.84–0.85/0.77–0.78). Pedipalp: trochanter 0.39/0.28–0.29 (0.43/0.28), femur 0.73–0.78/0.32–0.33 (0.78–0.80/0.35), patella 0.66–0.70/0.37–0.38 (0.73–0.74/0.39), chela (with pedicel) 1.26–1.33/0.47–0.50 (1.38–1.40/0.51), chela (without pedicel) 1.19–1.26 (1.30–1.31), hand (with pedicel) 0.76–0.81 (0.70), hand (without pedicel) 0.69–0.74 (0.77), movable finger length 0.51–0.60 (0.55–0.58). Leg I: trochanter 0.15–0.19/0.14 (0.16–0.17/0.15), femur 0.24–0.26/0.18–0.22 (0.25–0.27/0.20), patella 0.39–0.42/0.17–0.19 (0.44/0.20), tibia 0.35–0.39/0.12–0.13 (0.40–0.41/0.13), tarsus 0.33/0.09–0.10 (0.34–0.35/0.09). Leg IV: trochanter 0.28–0.30/0.16–0.17 (0.31–0.33/0.21), femoropatella 0.83–0.86/0.31–0.32 (0.91/0.34), tibia 0.54–0.55/0.18 (0.59–0.60/0.20), tarsus 0.38–0.39/0.12 (0.42/0.13).

#### Diagnosis

This new species is characterised by (see taxon discussion for more details): with smaller body size (3.39–3.49 (♂) mm, 3.17–3.26 (♀) mm); anterior half of carapace darker than posterior half; with two distinct eyespots; palpal femur 2.28–2.36 (♂), 2.23–2.29 (♀), chela with pedicel 2.66–2.68 (♂), 2.71–2.75 (♀) × longer than broad, chela without pedicel 2.43–2.52 (♂), 2.55–2.57 (♀) × longer than broad; prolateral surface of femur and patella granular; distance between *est* and *esb* further than that of *ist* and *isb*; galea simple, short and simple branch.

#### Etymology

The specific name is derived from the Latin adjective *parvus* (-a, -um), meaning small and referring to the characters of smaller body size.

#### Distribution

China (Yunnan) (Fig. 16).

#### Taxon discussion

*Paratemnoidesparvus* sp. nov. is similar to *P.guangdongensis* sp. nov., but differs by stouter chela (♂) (e.g. chela with pedicel 2.54–2.66× vs. 2.73× longer than broad) and fewer movable chelal finger teeth (♂) (41–42 vs. 54–56) .

*Paratemnoidesparvus* sp. nov. can be distinguished from *P.assimilis* by the number of posterior margin setae on the carapace (7–8 vs. 6), the number of serrula exterior blades (22–26 vs. 21) and slightly stouter pedipalps (♂) (e.g. chela with pedicel 2.66–2.68× vs. 2.60× longer than broad; chelal hand with pedicel 1.62× vs. 1.88× longer than broad, length 0.76–0.81 mm vs. 0.94 mm); from *P.borneoensis* by smaller body size and the slender pedipalps (♀) (e.g. body length 3.17–3.26 mm vs. 3.50 mm; palpal femur length 0.78–0.80 mm vs. 0.68 mm); from *P.curtulus* by the arrangement of trichobothria (e.g. distance between *est* and *esb* further than that of *ist* and *isb* vs. shorter than that of *ist* and *isb*) and the slender chela (♂) (chela with pedicel 2.66–2.68× vs. 2.19× longer than broad, length 1.26–1.33 mm vs. 1.05 mm); from *P.indicus* by smaller body size and slender pedipalps (e.g. body length (♂) 3.39–3.49 mm vs. 4.02 mm, (♀) 3.17–3.26 mm vs. 3.50 mm; palpal femur (♀) 2.23–2.29× vs. 2.00× longer than broad, length 0.78–0.80 mm vs. 0.72 mm) and the trait of eyespots (with two distinct eyespots vs. eyespots absent); from *P.japonicus* by the trait of eyes (with two distinct eyespots vs. eyespots absent), the number of serrula exterior blades (22–26 vs. 18) and larger body size and stouter pedipalps (♂) (e.g. body length 3.39–3.49 mm vs. 2.97 mm; palpal femur 2.28–2.36× vs. 2.40× longer than broad, length 0.73–0.78 mm vs. 0.81 mm); from *P.laosanus* by smaller body length (♂♀) (3.17–3.49 mm vs. 3.50–4.00 mm) and stouter chela (♂) (e.g. chela without pedicel 2.43–2.52× vs. 2.20–2.30× longer than broad; hand with pedicel 1.62× vs. 1.70–1.80× longer than broad, length 0.76–0.81 mm vs. 0.95 mm); from *P.mahnerti* by the number of serrula exterior blades (22–26 vs. 19) and relative position of trichobothrium *st* (*st* situated closer to *sb* than to *t* vs. midway between *sb* and *t*); from *P.pallidus* by smaller body size (♀) (e.g. body length 3.17–3.26 mm vs. 4.30 mm) and slender chela (♀) (e.g. chela with pedicel 2.71–2.75× vs. 2.20× longer than broad); from *P.philippinus* by slightly smaller body size and stouter chela (♂) (e.g. chela with pedicel 2.66–2.68× vs. 2.20× longer than broad; chelal hand with pedicel length 0.76–0.81 mm vs. 0.90 mm) and the trait of eyespots (with two distinct eyespots vs. without eyespots); from *P.plebejus* by smaller body size (♀) (e.g. body length 3.17–3.26 mm vs. 4.00–5.70 mm; palpal femur 2.23–2.29× vs. 2.00× longer than broad) and the trait of pedipalp (prolateral surface of femur and patella granular only vs. almost entire surface granular); from *P.pococki* by smaller body size and slender pedipalps (♀) (e.g. body length 3.17–3.26 mm vs. 3.87 mm; palpal femur length 0.78–0.80 mm vs. 0.62 mm; movable chelal finger length 0.55–0.58 mm vs. 0.46 mm); from *P.politus* sp. nov. by slender pedipalps (♂) (e.g. palpal femur 2.11–2.36× vs. 1.94–2.00× longer than broad) and fewer movable chelal finger teeth (♂) (41–42 vs. 48–50) ; from *P.redikorzevi* by the number of serrula exterior blades (22–26 vs. 20) and slightly larger body size and slender pedipalps (e.g. body length (♂) 3.39–3.49 mm vs. 3.00 mm; palpal femur (♀) 2.23–2.29× vs. 2.18–2.19× longer than broad, length 0.78–0.80 mm vs. 0.70–0.72 mm); from *P.robustus* by smaller body length (e.g. body length (♂) 3.39–3.49 mm vs. 3.80 mm, (♀) 3.17–3.26 mm vs. 4.60 mm; palpal femur (♂) length 0.73–0.78 mm vs. 0.91 mm; movable finger length 0.51–0.60 mm vs. 0.66 mm) and stouter leg IV (e.g. femoropatella 2.68–2.69× vs. 2.40× longer than deep; tibia 3.00–3.06× vs. 3.30× longer than deep); from *P.salomonis* by the trait of eyespots (with two distinct eyespots vs. eyespots wanting), slightly smaller body length and the slender pedipalps (♂) (e.g. palpal femur 2.28–2.36× vs. 2.10× longer than broad, length 0.73–0.78 mm vs. 0.69 mm; chela with pedicel 2.66–2.68× vs. 2.60× longer than broad; chela without pedicel 2.43–2.52× vs. 2.40× longer than broad); from *P.sinensis* by smaller body size (e.g. palpal patella (♂) 1.78–1.84× vs. 1.90× longer than broad, length 0.66–0.70 mm vs. 0.74–0.80 mm; tibia of leg IV (♂) 3.00–3.06× vs. 3.20× longer than deep) and the arrangement of trichobothria (distance between *est* and *esb* further than that of *ist* and *isb* vs. nearly equal to that of *ist* and *isb*); from *P.sumatranus* by larger body size (♂) (e.g. body length 3.39–3.49 mm vs. 2.50 mm; palpal femur length 0.73–0.78 mm vs. 0.61 mm; chela with pedicel 2.66–2.68× vs. 2.80–2.90× longer than broad; movable chelal finger length 0.51–0.60 mm vs. 0.47 mm); from *P.trisulcus* sp. nov. by stouter chela (♂) (e.g. chela with pedicel 2.54–2.66× vs. 2.83× longer than broad) and fewer movable chelal finger teeth (♂) (41–42 vs. 46) ; from *P.yunnanensis* sp. nov. by fewer movable chelal finger teeth (♂) (41–42 vs. 46–48) and relative position of trichobothrium *ist* (i.e. *ist* situated basal to *est* vs. distal to to *est*) (With 1906, With 1907, Beier 1932, Beier 1935a, Beier 1935b, Redikorzev 1938, Beier 1951, Morikawa 1953, Sivaraman 1980, Sivaraman 1981, Mathew and Joseph 2019).

### Paratemnoides politus

Hou, Zhao & Zhang sp. nov.

7E3A31BB-A989-517B-8836-59BAF4CA05B3

C58CD7D7-59F3-4A54-91BE-F283BFCE12D7

#### Materials

- **Type status:** Holotype. **Occurrence:** recordedBy: Chi Jin; individualCount: 1; sex: male; lifeStage: adult; occurrenceID: 94E993C0-3DAE-501B-9753-EA39FF342ED6; **Taxon:** scientificName: *Paratemnoidespolitus*; **Location:** country: China; stateProvince: Yunnan; county: Mengla; locality: Menglun Town, Xishuangbanna Tropical Botanical Garden; verbatimElevation: 605 m; verbatimCoordinates: 21°54.552′N, 101°16.831′E; **Event:** year: 2018; month: 7; day: 13; **Record Level:** institutionID: the Museum of Hebei University (MHBU); institutionCode: MHBU-YNBN180601
- **Type status:** Paratype. **Occurrence:** recordedBy: Chi Jin; individualCount: 1; sex: 1 male; lifeStage: adult; occurrenceID: E933A3C0-F822-5B0A-BC2D-316D99C7757D; **Taxon:** scientificName: *Paratemnoidespolitus*; **Location:** country: China; stateProvince: Yunnan; county: Mengla; locality: Menglun Town, Xishuangbanna Tropical Botanical Garden; verbatimElevation: 605 m; verbatimCoordinates: 21°54.552′N, 101°16.831′E; **Event:** year: 2018; month: 7; day: 13; **Record Level:** institutionID: the Museum of Hebei University (MHBU); institutionCode: MHBU-YNBN180602
- **Type status:** Paratype. **Occurrence:** recordedBy: Chi Jin; individualCount: 13; sex: 13 females; lifeStage: adult; occurrenceID: BA6D9A9B-7CAD-533A-9EA0-0250EE7AF89A; **Taxon:** scientificName: *Paratemnoidespolitus*; **Location:** country: China; stateProvince: Yunnan; county: Mengla; locality: Menglun Town, Xishuangbanna Tropical Botanical Garden; verbatimElevation: 605 m; verbatimCoordinates: 21°54.552′N, 101°16.831′E; **Event:** year: 2018; month: 7; day: 13; **Record Level:** institutionID: the Museum of Hebei University (MHBU); institutionCode: MHBU-YNBN180603–15

#### Description

**Figure 7.**
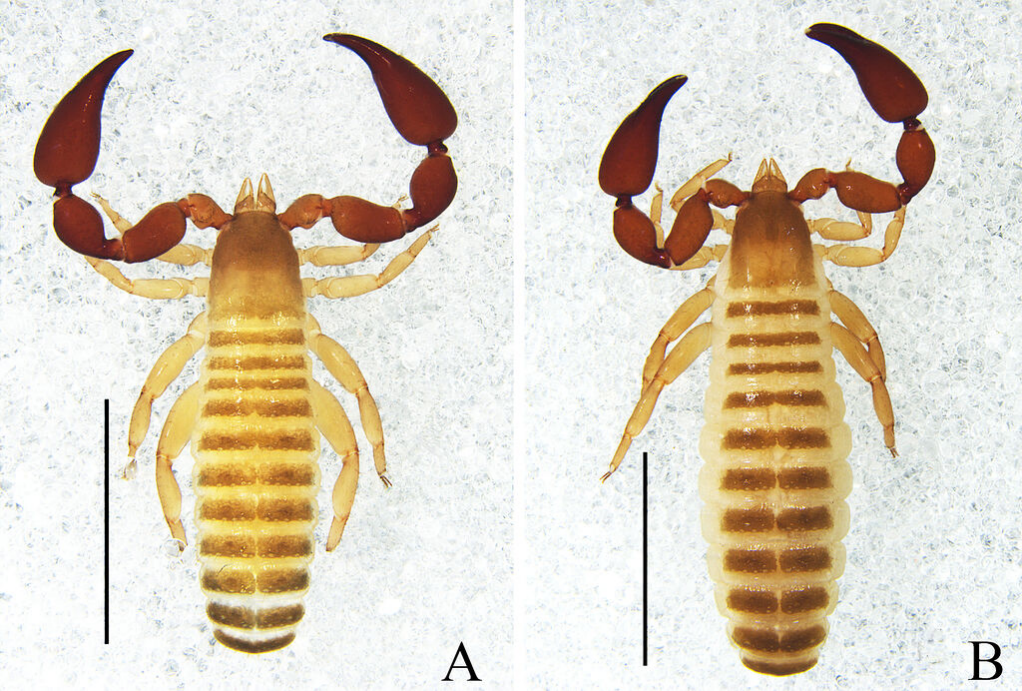
*Paratemnoidespolitus* sp. nov. **A** holotype male, habitus (dorsal view); **B** paratype female, habitus (dorsal view). Scale bars: 2.00 mm.

**Figure 8.**
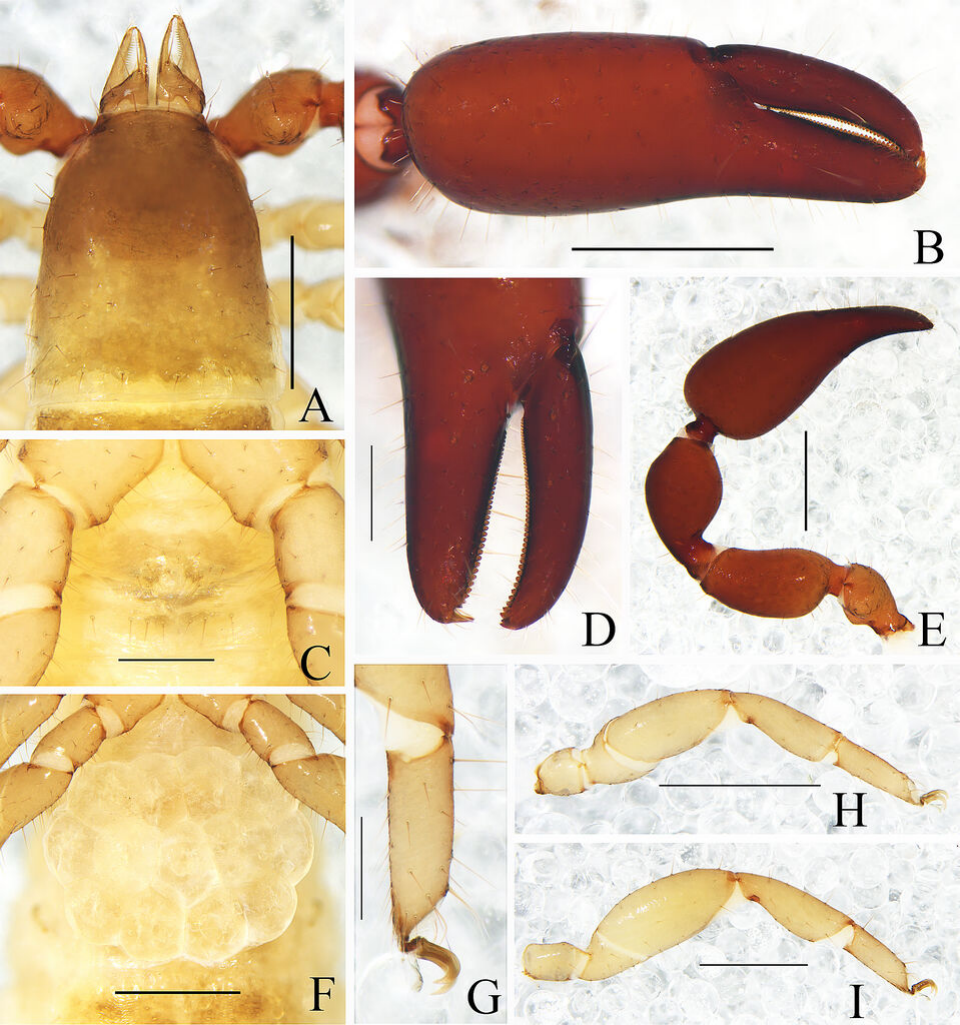
*Paratemnoidespolitus* sp. nov., holotype male (**A–E, G–I**), paratype female (**F**) **A** carapace (dorsal view); **B** left chela (lateral view); **C** male genital area (ventral view); **D** left chelal fingers (lateral view); **E** left pedipalp (dorsal view); **F** female genital area (ventral view), with eggs; **G** tarsus of left leg IV (lateral view); **H** left leg I (lateral view); **I** left leg IV (lateral view). Scale bars: 0.50 mm (**A, B, E, F, H, I**); 0.20 mm (**C, D, G**).

**Figure 9.**
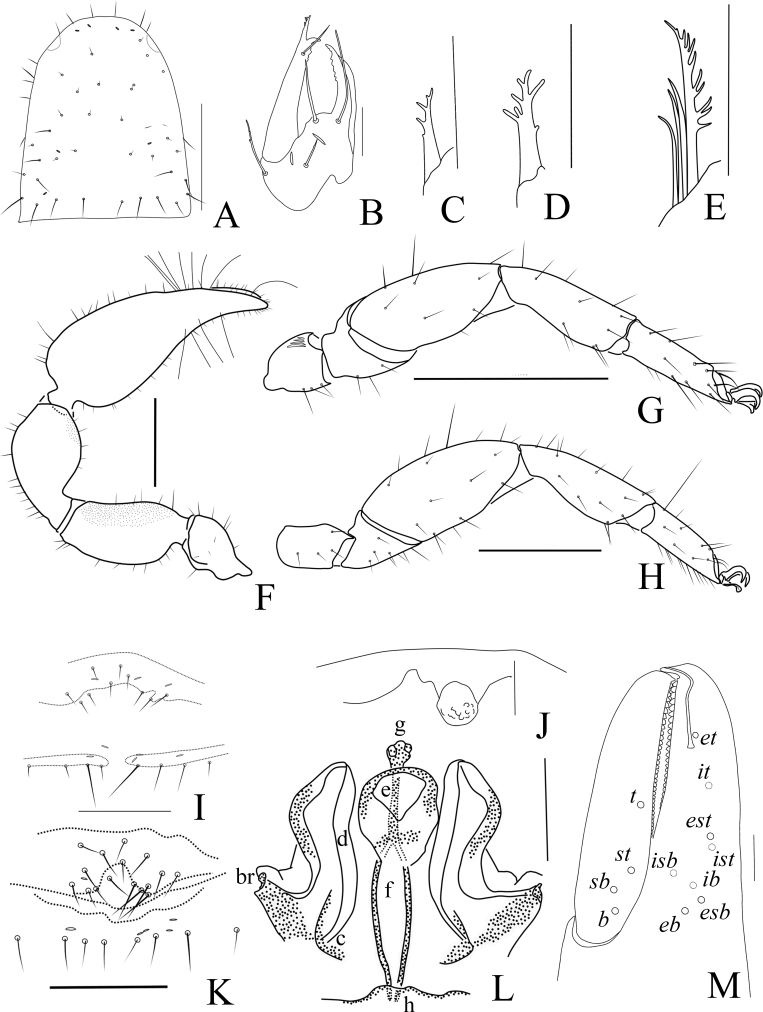
*Paratemnoidespolitus* sp. nov., holotype male (**A–C, E–H, K–M**), paratype female (**D, I, J**) **A** carapace (dorsal view); **B** left chelicera (dorsal view); **C** male galea; **D** female galea; **E** rallum; **F** left pedipalp (dorsal view); **G** left leg I (lateral view); **H** left leg IV (lateral view); **I** female genital area (ventral view); **J** spermatheca; **K** male genital area (ventral view); **L** male genital organ; **M** left chelal fingers (lateral view), with details of trichobothrial pattern. Scale bars: 0.50 mm (**A, F–H**); 0.20 mm (**I, K**); 0.10 mm (**B–E, J, L, M**).

Males (holotype and paratype) (Fig. 7A, Fig. 8A–E, G–I, Fig. 9A–C, E–H and K–M). Colour: anterior half of carapace, tergites and chelicerae brown, but paler in posterior half of carapace; pedipalps reddish-brown, paler and paler from fingertips to trochanter; remainder yellowish-brown.

Carapace (Figs 8, 9A): 1.16–1.18× longer than broad; surface smooth, without furrow; anterior half slightly darker than posterior half; with two distinct eyespots situated near anterior margin of carapace; anterior margin with six setae, posterior margin with nine setae, 52 in total, each seta acicular and very slightly curved.

Chelicera (Fig. 9B–C and E): much smaller than carapace length; surface smooth; four setae (*sbs* absent; *bs* shorter than others) and two lyrifissures (exterior condylar lyrifissure and exterior lyrifissure) present on hand; movable finger with one slightly curved galeal seta; *bs* and *es* dentate apically, *is* and *ls* long and acute. Fixed finger with four large retrorse teeth and four small apical teeth, movable finger with a long broadly dentated subapical lobe and one small sub-terminal tooth; galea present, shorter and with three larger lateral branchlets and one small terminal dentation (Fig. 9C). Serrula interior connected to fixed finger for entire length, proximally modified to form velum, serrula exterior with 22 blades, the basal one longest; lamina exterior present. Rallum composed of four blades, the basal two blades shorter than others, the distal one dentated anteriorly, remainder smooth (Fig. 9E).

Pedipalp (Fig. 8B, D–E, Fig. 9F and M): stout, trochanter 1.46–1.50×, femur (base distinctly wider than terminal) 1.94–2.00×, patella 1.80–1.93×, chela with pedicel (without pedicel) 2.67–2.87× (2.43–2.62×), hand with pedicel (without pedicel) 1.65–1.77× (1.41–1.52×) longer than broad; movable chelal finger 0.67–0.69× (0.78–0.81×) longer than hand with pedicel (without pedicel) and 0.42–0.43× (0.46–0.47×) longer than chela with pedicel (without pedicel). Setae generally long and acuminate. Prolateral surface of femur and patella light yellow and granular; trochanter with one well-developed conical dorsal tubercle and a rounded ventral tubercle. Fixed chelal finger with eight trichobothria, movable chelal finger with four trichobothria: *eb* and *esb* situated at base of fixed finger on retrolateral face, *esb* slightly distal to *eb*; *ib* and *isb* situated at base of fixed finger on prolateral face, *isb* slightly distal to *ib*; *est* in the middle of fixed finger; *et* near sub-distal of fixed finger; *est* closer to *esb* than to *et*; *it* distal to *est* and proximal to *et*; *ist* slightly proximal to *est*; *it* closer to *ist* than to fingertip; distance between *est* and *esb* further than that of *ist* and *isb*; distance between *it* and fingertip further than distance between *ist* and *isb*; *b* and *sb* situated at base of movable finger on retrolateral face; *t* in the middle of movable finger and at same level as *it*; *sb* slightly closer to *b* than to *st*; *st* closer to *sb* than to *t* (Fig. 9M). Venom apparatus only present in fixed chelal finger, venom ducts curved and short, terminating in inflated nodus ramosus between *et* and *est*, very close to *et.* Both chelal fingers with a row of acute teeth, spaced contiguously along the margin, slightly rounded proximally: fixed chelal finger with 37–38 teeth; movable chelal finger with 48–50 teeth (nearly as large as teeth on fixed chelal finger); without accessory teeth (Fig. 9M). Femur without long tactile setae. Movable chelal finger straight in lateral view (Fig. 8D and Fig. 9M).

Opisthosoma: generally typical, all setae long, acuminate and biseriate; pleural membrane longitudinally striate, without setae. Tergites I–V and XI undivided and others incompletely divided, each anterior half of tergite darker than posterior half, tergal chaetotaxy I–XI: 10: 11: 12: 13: 14: 7–7: 8–7: 8–7: 7–7: 9–8: 7–6, tergites X and XI each with two long tactile setae. All sternites (except sternite XI) divided, sternal chaetotaxy IV–XI: 5–5: 9–9: 8–7: 8–8: 8–8: 7–8: 9–8 (2T): 8–7 (4T). Anus (tergite Ⅻ and sternite Ⅻ) without raised rim. Anterior genital operculum with 17 setae on each side, posterior margin with eight setae. Male genitalia (Fig. 8C, Fig. 9K and L): lateral apodemes (a) relatively small; the hooked branch (br) well-developed, bowed distally and terminating in a plate-like tip; the proximal part with a nearly pale sclerotised bar (c), distinctly curved; the longitudinal fold of medial diverticula (d) vestigial; the ejaculatory canal atrium (e) not well-developed, curved distally; the lateral rods (f) short and diverging proximally; the tip of dorsal apodeme (g) completely joined; the ventral diverticulum (h) bilobed; genital atrium without genital setae.

Legs (Fig. 8G–I and Fig. 9G–H): generally typical, fairly smooth, slightly stout; junction between femora and patellae I and II oblique. Femoropatella of leg Ⅳ 2.74–2.82× longer than deep; tibia 3.00× longer than deep; with basal tactile setae on tarsal segment: tarsus 2.92–3.07× longer than deep (TS = 0.12–0.13); subterminal tarsal setae arcuate and acute. Arolium slightly shorter than claws, not divided; claws smooth.

Adult females (Fig. 7B, Fig. 8F, Fig. 9D and I–J): Mostly same as the males, but a little larger and darker. Carapace: 1.12–1.13× longer than broad; anterior margin and posterior margin each with six setae, 62 in total. Chelicera: hand with four setae; galea with four larger branchlets and one small branchlet; serrula exterior with 23 blades, the basal one longest; rallum composed of four blades, the distal one dentated anteriorly, remainder smooth. Pedipalps: stout, trochanter 1.48–1.54, femur 1.92–2.00, patella 1.92, chela (with pedicel) 2.71–2.72, chela (without pedicel) 2.47–2.48, hand (without pedicel) 1.51× longer than broad, movable chelal finger 0.68–0.69× (0.79–0.81×) longer than hand with pedicel (without pedicel) and 0.42× (0.46–0.47×) longer than chela with pedicel (without pedicel). Opisthosoma: female genitalia: simple, spermathecae provided with separated median cribriform plates. Legs: femoropatella of leg Ⅳ 2.71–2.74× longer than deep; tibia 2.81–2.86× longer than deep; with basal tactile setae on tarsalc segment: tarsus 2.93–3.31× longer than deep (TS = 0.12).

Dimensions (length/breadth or, in the case of the legs, length/depth in mm; ratios in parentheses). Males (females in parentheses): body length 3.31–3.63 (4.58–4.65). Carapace 0.97–1.00/0.82–0.86 (1.03/0.91–0.92). Pedipalp: trochanter 0.41–0.45/0.28–0.30 (0.43/0.28–0.29), femur 0.68–0.78/0.35–0.39 (0.71–0.72/0.36–0.37), patella 0.72–0.79/0.40–0.41 (0.73–0.75/0.38–0.39), chela (with pedicel) 1.36–1.49/0.51–0.52 (1.41–1.44/0.52–0.53), chela (without pedicel) 1.24–1.36 (1.29–1.31), hand (with pedicel) 0.84–0.92 (0.87–0.88), hand (without pedicel) 0.72–0.79 (0.75), movable finger length 0.58–0.62 (0.59–0.61). Leg I: trochanter 0.17–0.18/0.15–0.16 (0.17–0.19/0.16), femur 0.26–0.28/0.20–0.22 (0.27–0.28/0.21–0.22), patella 0.41–0.44/0.18–0.19 (0.43–0.45/0.18–0.19), tibia 0.37–0.38/0.13 (0.39–0.40/0.14), tarsus 0.30–0.31/0.09–0.10 (0.33/0.10). Leg IV: trochanter 0.32/0.18–0.22 (0.33/0.20–0.22), femoropatella 0.85–0.93/0.31–0.33 (0.92–0.96/0.34–0.35), tibia 0.60–0.63/0.20–0.21 (0.59–0.60/0.21), tarsus 0.38–0.43/0.13–0.14 (0.41–0.43/0.13–0.14).

#### Diagnosis

This new species is characterised by (see taxon discussion for more details): carapace 1.16–1.18 (♂), 1.12–1.13 (♀) × longer than broad; with two distinct eyespots; palpal femur 1.94–2.00 (♂), 1.92–2.00 (♀), chela with pedicel 2.67–2.87 (♂), 2.71–2.72 (♀) × longer than broad, chela without pedicel 2.43–2.62 (♂), 2.47–2.48 (♀) × longer than broad; only prolateral surface of femur and patella granular; fixed chelal finger with 37–38 teeth; movable chelal finger with 48–50 teeth; serrula exterior with (♂♀) 22–23 blades.

#### Etymology

The specific name is derived from the Latin adjective *polio* (-a, -um), meaning smooth and referring to the characters of smooth pedipalps, only prolateral surface of femur and patella granular.

#### Distribution

China (Yunnan) (Fig. 16).

#### Taxon discussion

*Paratemnoidespolitus* sp. nov. is similar to *P.trisulcus* sp. nov., but differs by stouter pedipalps (♂) (e.g. palpal femur 1.94–2.00× vs. 2.39× longer than broad).

*Paratemnoidespolitus* sp. nov. can be distinguished from *P.assimilis* by slightly stouter appendages (♂) (e.g. chela with pedicel 2.67–2.87× vs. 2.60× longer than broad; chelal hand with pedicel 1.65–1.77× vs. 1.88× longer than broad; femoropatella of leg IV 2.71–2.74× vs. 2.50–2.60×, tibia of leg IV 2.81–2.86× vs. 3.00× longer than deep); from *P.borneoensis* by the number of serrula exterior blades (22 vs. 24), larger body size and stouter pedipalps (♀) (e.g. body length 4.58–4.65 mm vs. 3.50 mm; palpal femur 1.92–2.00× vs. 2.27× longer than broad, length 0.71–0.72 mm vs. 0.68 mm); from *P.curtulus* by the arrangement of trichobothria (e.g. distance between *est* and *esb* further than that of *ist* and *isb* vs. shorter to that of *ist* and *isb*) and the slender chela (♂) (chela with pedicel 2.67–2.87× vs. 2.19× longer than broad, length 1.36–1.49 mm vs. 1.05 mm); from *P.guangdongensis* sp. nov. by stouter pedipalps (♂) (e.g. palpal femur 1.94–2.00× vs. 2.20× longer than broad) and fewer movable chelal finger teeth (♂) (48–50 vs. 54–56); from *P.indicus* by the trait of eyespots (with two distinct eyespots vs. eyespots absent), the number of setae on the carapace (52 vs. 46), more movable chelal finger teeth (48–50 vs. 42) and the body size (e.g. body length (♂) 3.31–3.63 mm vs. 4.02 mm, (♀) 4.58–4.65 mm vs. 4.06 mm); from *P.japonicus* by the trait of eyes (with two distinct eyespots vs. eyespots absent), the number of serrula exterior blades (22 vs. 18) and larger body size and stouter pedipalps (♂) (e.g. body length 3.31–3.63 mm vs. 2.97 mm; palpal femur 1.94–2.00× vs. 2.40× longer than broad, length 0.68–0.78 mm vs. 0.81 mm); from *P.laosanus* by larger body size and stouter pedipalps (e.g. body length (♀) 4.58–4.65 mm vs. max. 4.00 mm; palpal femur length (♀) 0.71–0.72 mm vs. 0.77 mm; hand with pedicel (♀) 1.66–1.67× vs. 1.79× longer than broad, length 0.87–0.88 mm vs. 1.00 mm; chela without pedicel (♂) 2.43–2.62× vs. 2.20–2.30× longer than broad) and more chelal fingers teeth (fixed finger with 37–38 vs. 33 teeth, movable finger with 48–50 vs. 44); from *P.mahnerti* by the number of serrula exterior blades (22 vs. 19), relative position of trichobothrium *st* (*st* situated closer to *sb* than to *t* vs. midway between *sb* and *t*), more chelal fingers teeth (fixed finger with 37–38 vs. 31 teeth, movable finger with 48–50 vs. 39), the number of setae on the carapace (52 vs. 40) and larger body size (e.g. body length (♂) 3.31–3.63 mm vs. 2.02 mm, (♀) 4.58–4.65 mm vs. 3.05 mm); from *P.pallidus* by slightly larger body size (♀) (e.g. body length 4.58–4.65 mm vs. 4.30 mm), stouter pedipalps (♀) (e.g. palpal femur 1.92–2.00× vs. 2.20× longer than broad) and the number of serrula exterior blades (22 vs. 25); from *P.parvus* sp. nov. by stouter pedipalps (♂) (e.g. palpal femur 1.94–2.00× vs. 2.11–2.36× longer than broad) and more movable chelal finger teeth (♂) (48–50 vs. 41–42) ; from *P.philippinus* by the slender chela (♂) (chela with pedicel 2.67–2.87× vs. 2.20× longer than broad), the trait of eyespots (with two distinct eyespots vs. without eyespots) and the number of serrula exterior blades (22 vs. 26); from *P.plebejus* by stouter pedipalps (♂) (e.g. palpal femur 1.94–2.00× vs. 2.11× longer than broad), more fixed chelal finger teeth (♂) (37–38 vs. 32) and the trait of pedipalp (prolateral surface of femur and patella granular only vs. almost entire surface granular); from *P.pococki* by larger body size and slender pedipalps (♀) (e.g. body length 4.58–4.65 mm vs. 3.87 mm; palpal femur 1.92–2.00× vs. 2.20× longer than broad, length 0.71–0.72 mm vs. 0.62 mm; movable chelal finger length 0.59–0.61 mm vs. 0.46 mm); from *P.redikorzevi* by the number of serrula exterior blades (22 vs. 20), larger body size and slender pedipalps (e.g. body length (♂) 3.39–3.49 mm vs. 3.00 mm, (♀) 4.58–4.65 mm vs. 3.00–4.00 mm; chela without pedicel (♂) 2.43–2.62× vs. 2.30–2.40× longer than broad); from *P.robustus* by the number of serrula exterior blades (22 vs. 26), smaller body size and stouter pedipalps (♂) (e.g. body length 3.31–3.63 mm vs. 3.80 mm; palpal femur 1.94–2.00× vs. 2.28× longer than broad, length 0.68–0.78 mm vs. 0.91 mm); from *P.salomonis* by the trait of eyespots (with two distinct eyespots vs. eyespots wanting), the number of serrula exterior blades (22 vs. 23) and slightly slender appendages (e.g. chela with pedicel 2.67–2.87× vs. 2.60× longer than broad; tarsus of leg IV 2.92–3.07× vs. 3.20× longer than deep); from *P.sinensis* by stouter pedipalps (e.g. palpal femur (♂) 1.94–2.00× vs. 2.30× longer than broad, chela with pedicel (♂) 2.67–2.87× vs. 2.40–2.50× longer than broad) and the arrangement of trichobothria (distance between *est* and *esb* further than that of *ist* and *isb* vs. nearly equal to that of *ist* and *isb*); from *P.sumatranus* by the number of serrula exterior blades (22 vs. 24) and larger body size and stouter pedipalps (♂) (e.g. body length 3.31–3.63 mm vs. 2.50 mm; palpal femur 1.94–2.00× vs. 2.26× longer than broad, length 0.68–0.78 mm vs. 0.61 mm; movable chelal finger length 0.58–0.62 mm vs. 0.47 mm); from *P.yunnanensis* sp. nov. by stouter pedipalps (♂) (e.g. palpal femur 1.94–2.00× vs. 2.17–2.41× longer than broad) and relative position of trichobothria *t* and *it* (*t* distal to *it* vs. basal to *it*) (With 1906, With 1907, Beier 1932, Beier 1935a, Beier 1935b, Redikorzev 1938, Beier 1951, Morikawa 1953, Sivaraman 1980, Sivaraman 1981, Mathew and Joseph 2019).

### Paratemnoides trisulcus

Hou, Zhao & Zhang sp. nov.

263384BF-15CB-545E-A77A-B0C6CEAD8F17

E186E02C-29EC-4E22-83E0-58EAE4372331

#### Materials

- **Type status:** Holotype. **Occurrence:** recordedBy: Yannan Mu; individualCount: 1; sex: male; lifeStage: adult; occurrenceID: AF8FFA93-0F5C-5E0F-B7EE-C3A02A2B0772; **Taxon:** scientificName: *Paratemnoidestrisulcus*; **Location:** country: China; stateProvince: Guangxi; county: Huanjiang; verbatimElevation: 225 m; verbatimCoordinates: 24°49.675′N, 108°16.455′E; **Event:** year: 2019; month: 8; day: 3; **Record Level:** institutionID: the Museum of Hebei University (MHBU); institutionCode: MHBU-GXHJ19080301
- **Type status:** Paratype. **Occurrence:** recordedBy: Yannan Mu; individualCount: 3; sex: 3 females; lifeStage: adult; occurrenceID: A2C74D4B-6545-5B09-AD15-E31C4F76EB3C; **Taxon:** scientificName: *Paratemnoidestrisulcus*; **Location:** country: China; stateProvince: Guangxi; county: Huanjiang; verbatimElevation: 225 m; verbatimCoordinates: 24°49.675′N, 108°16.455′E; **Event:** year: 2019; month: 8; day: 3; **Record Level:** institutionID: the Museum of Hebei University (MHBU); institutionCode: MHBU-GXHJ19080302–04

#### Description

**Figure 10.**
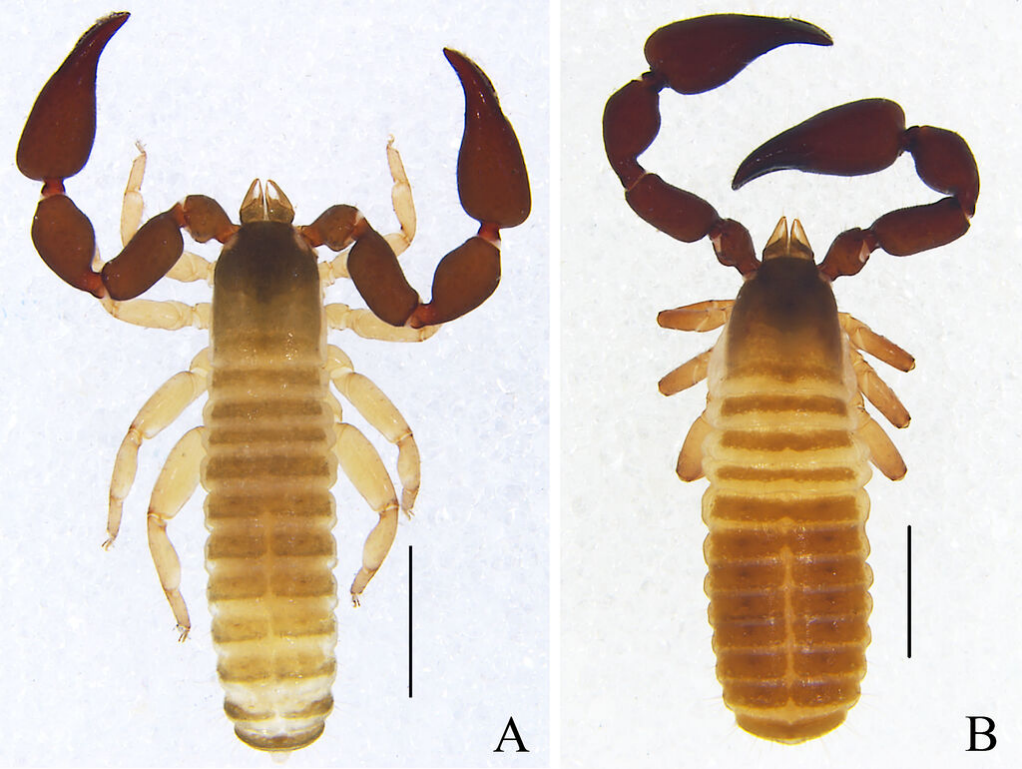
*Paratemnoidestrisulcus* sp. nov. **A** holotype male, habitus (dorsal view); **B** paratype female, habitus (dorsal view). Scale bars: 1.00 mm.

**Figure 11.**
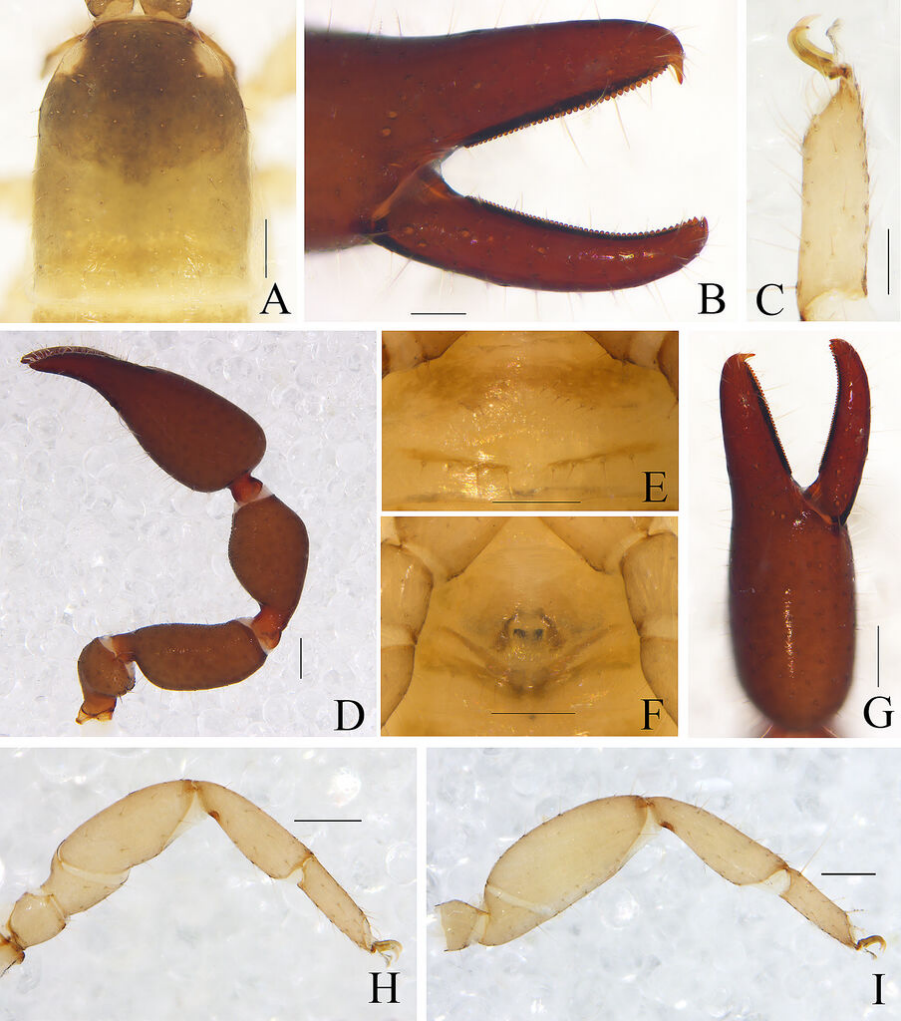
*Paratemnoidestrisulcus* sp. nov., holotype male (**A–D, F–I**), paratype female (**E**) **A** carapace (dorsal view); **B** right chelal fingers (lateral view); **C** tarsus of left leg IV (lateral view); **D** right pedipalp (dorsal view); **E** female genital area (ventral view); **F** male genital area (ventral view); **G** right chela (lateral view); **H** left leg I (lateral view); **I** left leg IV (lateral view). Scale bars: 0.20 mm (**A, D–I**); 0.10 mm (**B, C**).

**Figure 12.**
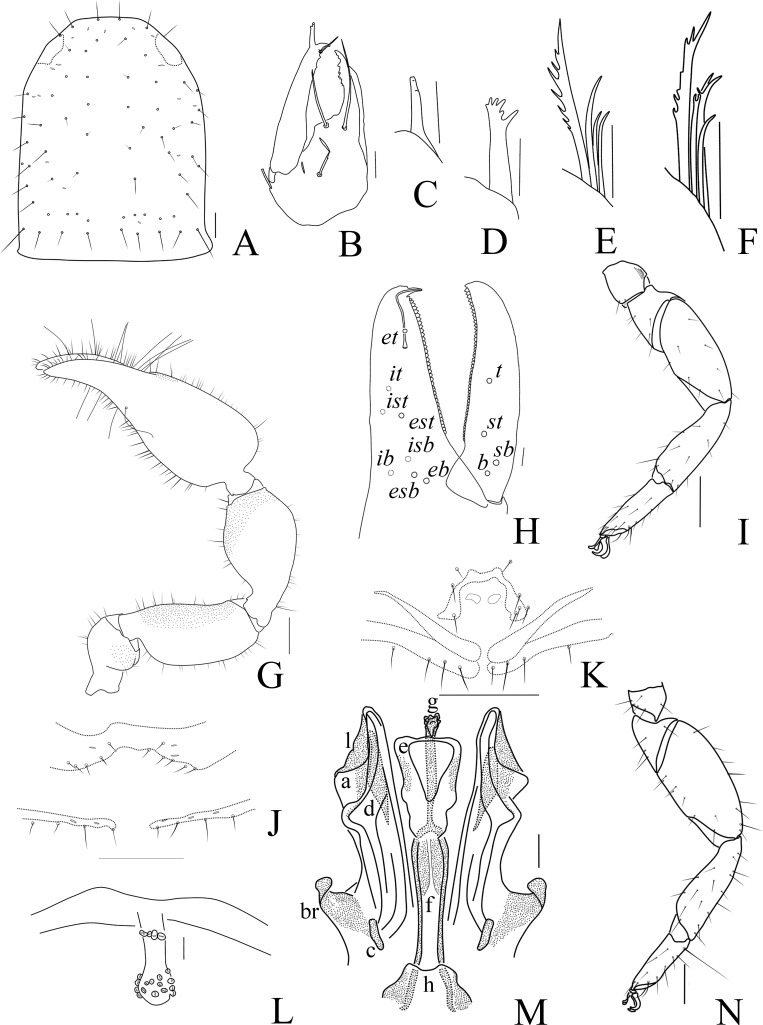
*Paratemnoidestrisulcus* sp. nov., holotype male (**A–C, E, G–I, K, M, N**), paratype female (**D, F, J, L**) **A** carapace (dorsal view); **B** left chelicera (dorsal view); **C** male galea; **D** female galea; **E** male rallum; **F** female rallum; **G** right pedipalp (dorsal view); **H** right chelal fingers (lateral view), with details of trichobothrial pattern; **I** left leg I (lateral view); **J** female genital area (ventral view); **K** male genital area (ventral view); **L** spermatheca; **M** male genital organ; **N** left leg IV (lateral view). Scale bars: 0.20 mm (**G, I–K, N**); 0.10 mm (**A–F, H, L, M**).

Male (holotype) (Fig. 10A, Fig. 11A–D, F–I, Fig. 12A–C, E, G–I, K and M–N). Colour: anterior half of carapace brown, but paler in posterior half; pedipalps reddish-brown; remainder yellowish-brown.

Carapace (Figs 11, 12A): 1.34× longer than broad; surface smooth, without furrow; anterior half slightly darker than posterior half; with two distinct eyespots situated near anterior margin of carapace; anterior margin with four setae, posterior margin with nine setae, 67 in total, each seta acicular and very slightly curved.

Chelicera (Fig. 12B–C and E): much smaller than carapace length; surface smooth; four setae (*sbs* absent; *bs* shorter than others) and two lyrifissures (exterior condylar lyrifissure and exterior lyrifissure) present on hand; movable finger with one slightly curved galeal seta; *bs* and *es* dentate apically, *is* and *ls* long and acute. Fixed finger with four large retrorse teeth and two small apical teeth, movable finger with a long broadly dentated subapical lobe; galea present, slender and with five small terminal dentations (Fig. 12C). Serrula interior connected to fixed finger for entire length, proximally modified to form velum, serrula exterior with 21 blades, the basal one longest; lamina exterior present. Rallum composed of four blades, the basal two blades shorter than others, the distal one dentated anteriorly, remainder smooth (Fig. 12E).

Pedipalp (Fig. 11B, D, G, Fig. 12G and H): stout, trochanter 1.53×, femur 2.39×, patella 1.76×, chela with pedicel (without pedicel) 2.83× (2.64×), hand without pedicel 1.49× longer than broad; movable chelal finger 0.72× longer than hand without pedicel and 0.43× (0.46×) longer than chela with pedicel (without pedicel). Setae generally long and acuminate. Retrolateral surface of trochanter, prolateral surface of femur, patella and base of chelal fingers granular; trochanter with two well-developed rounded tubercles. Fixed chelal finger with eight trichobothria, movable chelal finger with four trichobothria: *eb* and *esb* situated at base of fixed finger on retrolateral face, *esb* slightly distal to *eb*; *ib* and *isb* situated at base of fixed finger on prolateral face, *isb* slightly distal to *ib*; *est* in the middle of fixed finger; *et* near sub-distal of fixed finger; *est* closer to *esb* than to *et*; *it* distal to *est* and proximal to *et*; *ist* slightly distal to *est* and proximal to *it*; *it* closer to *ist* than to fingertip; distance between *est* and *esb* further than that of *ist* and *isb*; distance between *it* and fingertip further than distance between *ist* and *isb*; *b* and *sb* situated at base of movable finger on retrolateral face; *t* in the middle of movable finger and at same level as *it*; *sb* slightly closer to *b* than to *st*; *st* closer to *sb* than to *t* (Fig. 12H). Venom apparatus only present in fixed chelal finger, venom ducts curved and short, terminating in inflated nodus ramosus between *et* and *est*, closer to *et.* Both chelal fingers with a row of acute teeth, spaced contiguously along the margin, slightly rounded proximally: fixed chelal finger with 38 teeth; movable chelal finger with 46 teeth (nearly as large as teeth on fixed chelal finger); without accessory teeth (Fig. 12H). Femur without long tactile setae. Movable chelal finger slightly curved in lateral view (Fig. 11B and Fig. 12H).

Opisthosoma: generally typical, all setae long, acuminate and biseriate; pleural membrane longitudinally striate, without setae. Tergites I–V and XI undivided and others incompletely divided, tergal chaetotaxy I–XI: 8: 9: 10: 14: 13: 7–7: 7–7: 6–7: 7–7: 6–6 (4T): 10 (2T). All sternites divided, sternal chaetotaxy IV–XI: 6–4: 7–7: 7–6: 7–8: 7–7: 7–7: 6–5 (4T): 12 (4T). Anus (tergite Ⅻ and sternite Ⅻ) without raised rim. Anterior genital operculum with three or five setae on each side, posterior margin with eight setae. Male genitalia (Fig. 11F, Fig. 12K and M) well-developed: lateral apodemes (a) relatively well-developed, with a distinctive inner ridge curved into semicircle; the hooked branch (br) well-developed, bowed distally and terminated in a plate-like tip; the proximal part with a dark sclerotised bar (c), distinctly curved; the longitudinal fold of medial diverticula (d) with a projection midway along its length; the ejaculatory canal atrium (e) not well-developed, curved distally; the lateral rods (f) short and diverging proximally; the tip of dorsal apodeme (g) completely joined; the ventral diverticulum (h) bilobed; genital atrium without genital setae.

Legs (Fig. 11C, H–I, Fig. 12I and N): generally typical, fairly smooth, slightly stout; junction between femora and patellae I and II oblique. Femoropatella of leg Ⅳ 2.61× longer than deep; tibia 3.17× longer than deep; with basal tactile setae on tarsal segment: tarsus 3.36× longer than deep (TS = 0.14); subterminal tarsal setae arcuate and acute. Arolium slightly shorter than claws, not divided; claws smooth.

Adult females (Fig. 10B, Fig. 11E, Fig. 12J and L): Mostly same as the male, but a little darker. Carapace: 1.04–1.12× longer than broad; anterior margin with six setae, posterior margin with nine setae, 69 in total. Chelicera: hand with four setae; galea with five branchlets; serrula exterior with 20 blades, the basal one longest; rallum composed of four blades, the distal three dentated anteriorly. Pedipalps: stout, trochanter 1.35–1.43, femur 2.17–2.20, patella 1.79, chela (with pedicel) 2.60–2.61, chela (without pedicel) 2.43–2.44, hand (without pedicel) 1.38–1.42× longer than broad, movable chelal finger 0.67–0.68× longer than hand without pedicel; fixed chelal finger with 35 teeth; movable chelal finger with 48 teeth. Opisthosoma: tergites I–IV and XI undivided and others incompletely divided, tergal chaetotaxy I–XI: 10: 10: 10: 13: 6–7: 7–7: 6–8: 8–7: 7–7: 6–7 (4T): 12 (2T). Sternites incompletely divided, sternal chaetotaxy IV–XI: 5–5: 8–8: 8–8: 8–7: 8–8: 8–7: 6–7 (4T): 9 (4T). Female genitalia: simple, spermathecae provided with separated median cribriform plates. Legs: femoropatella of leg Ⅳ 2.49–2.56× longer than deep; tibia 3.05–3.10× longer than deep; with basal tactile setae on tarsal segment: tarsus 3.00–3.15× longer than deep (TS = 0.12).

Dimensions (length/breadth or, in the case of the legs, length/depth in mm; ratios in parentheses). Male (females in parentheses): body length 3.47 (2.87–3.65). Carapace 0.98/0.73 (1.00–1.03/0.92–0.96). Pedipalp: trochanter 0.46/0.30 (3.20–3.30/0.92–0.96), femur 0.79/0.33 (0.77–0.78/0.35–0.36), patella 0.72/0.41 (0.75/0.42), chela (with pedicel) 1.33/0.47 (1.41–1.43/0.54–0.55), chela (without pedicel) 1.24 (1.31–1.34), hand (with pedicel) 0.88 (0.66–0.69), hand (without pedicel) 0.79 (0.75–0.78), movable finger length 0.57 (0.58). Leg I: trochanter 0.17/0.16 (0.17–0.18/0.16), femur 0.27/0.21(0.27–0.28/0.22–0.23), patella 0.46/0.20 (0.45–0.46/0.20–0.22), tibia 0.42/0.14 (0.42–0.43/0.14–0.15), tarsus 0.31/0.09 (0.32–0.33/0.10). Leg IV: trochanter 0.31/0.18 (0.32–0.33/0.18–0.20), femoropatella 0.81/0.31 (0.82–0.87/0.32–0.35), tibia 0.57/0.18 (0.61–0.62/0.20), tarsus 0.37/0.11 (0.39–0.41/0.13).

#### Diagnosis

This new species is characterised by (see taxon discussion for more details): carapace with two distinct eyespots; palpal femur 2.39 (♂), 2.17–2.20 (♀), chela with pedicel 2.83 (♂), 2.60–2.61 (♀) × longer than broad, chela without pedicel 2.64 (♂), 2.43–2.44 (♀) × longer than broad; retrolateral surface of trochanter, prolateral surface of femur and patella granular; female rallum with three dentated blades; male genitalia: distal part lateral apodemes (a) well-developed.

#### Etymology

The specific name is derived from the Latin adjective *trisulcus* (-a, -um), meaning trifurcate and referring to the characters of rallum (♀) with three dentate blades.

#### Distribution

China (Guangxi) (Fig. 16).

#### Taxon discussion

*Paratemnoidestrisulcus* sp. nov. is similar to *P.politus* sp. nov., but differs by slender pedipalps (♂) (e.g. palpal femur 2.39× vs. 1.94–2.00× longer than broad).

*Paratemnoidestrisulcus* sp. nov. can be distinguished from *P.assimilis* by the number of posterior margin setae on the carapace (9 vs. 6), the trait of rallum (♀) (rallum with three dentated blades vs. with one dentated blade only) and stouter pedipalps (♂) (e.g. chela with pedicel 2.83× vs. 2.60× longer than broad; palpal patella 1.76× vs. 2.00× longer than broad); from *P.borneoensis* by the number of serrula exterior blades (21 vs. 24) and the slender pedipalps (♀) (e.g. palpal femur length 0.77–0.78 mm vs. 0.68 mm; palpal patella 1.79× vs. 2.10× longer than broad, length 0.75 mm vs. 0.68 mm); from *P.curtulus* by the number of serrula exterior blades (21 vs. 23), the arrangement of trichobothria (e.g. distance between *est* and *esb* further than that of *ist* and *isb* vs. shorter than that of *ist* and *isb*) and the slender chela (♂) (chela with pedicel 2.83× vs. 2.19× longer than broad, length 1.33 mm vs. 1.05 mm); from *P.guangdongensis* sp. nov. by slender pedipalps (♂) (e.g. palpal femur 2.39× vs. 2.20× longer than broad); from *P.indicus* by smaller body size and slender pedipalps (e.g. body length (♂) 3.47 mm vs. 4.02 mm; palpal femur (♀) 2.17–2.20× vs. 2.00× longer than broad, length 0.77–0.78 mm vs. 0.72 mm) and the trait of eyespots (with two distinct eyespots vs. eyespots absent); from *P.japonicus* by the trait of eyes (with two distinct eyespots vs. eyespots absent), the number of serrula exterior blades (21 vs. 18) and larger body size and stouter pedipalps (♂) (e.g. body length 3.47 mm vs. 2.97 mm; palpal patella 1.76× vs. 1.90× longer than broad); from *P.laosanus* by slender pedipalps (♂) (e.g. palpal femur 2.39× vs. 1.97× longer than broad; chela without pedicel 2.64× vs. 2.20–2.30× longer than broad); from *P.mahnerti* by the number of serrula exterior blades (21 vs. 19), relative position of trichobothrium *st* (*st* situated closer to *sb* than to *t* vs. midway between *sb* and *t*), the number of setae on the carapace (67 vs. 40), larger body size (♂) (e.g. body length 3.47 mm vs. 2.02 mm) and stouter pedipalps (♀) (e.g., palpal femur 2.17–2.20× vs. 2.29× longer than broad, length 0.77–0.78 mm vs. 0.90 mm; chela with pedicel 2.60–2.61× vs. 2.90× longer than broad, length 1.41–1.43 mm vs. 1.62 mm; chela without pedicel 2.43–2.44× vs. 2.64× longer than broad, length 1.31–1.34 mm vs. 1.48 mm); from *P.pallidus* by the number of serrula exterior blades (21 vs. 25), smaller body size and slender chela (♀) (e.g. body length 2.87–3.65 mm vs. 4.30 mm; chela with pedicel 2.60–2.61× vs. 2.20× longer than broad); from *P.parvus* sp. nov. by slender chela (♂) (e.g. chela with pedicel 2.83× vs. 2.54–2.66× longer than broad) and more movable chelal finger teeth (♂) (46 vs. 41–42) ; from *P.philippinus* by the trait of eyespots (with two distinct eyespots vs. without eyespots), the number of serrula exterior blades (21 vs. 26) and slender appendages (♂) (chela with pedicel 2.83× vs. 2.20× longer than broad; femoropatella of leg IV 2.61× vs. 2.30× longer than deep); from *P.plebejus* by the trait of rallum (♂♀) (rallum with three dentated blades vs. with one dentated blade only), stouter pedipalps (♂♀) (e.g. palpal femur (♂) 2.39×, (♀) 2.17–2.20× vs. (♂) 2.11×, (♀) 2.00× longer than broad) and the trait of pedipalp (prolateral surface of femur and patella granular only vs. almost entire surface granular); from *P.pococki* by smaller body size and slender pedipalps (♀) (e.g. body length 2.87–3.65 mm vs. 3.87 mm; palpal femur length 0.77–0.78 mm vs. 0.62 mm; movable chelal finger length 0.58 mm vs. 0.46 mm); from *P.redikorzevi* by slightly larger body size and slender pedipalps (♂) (e.g. body length 3.47 mm vs. 3.00 mm; palpal femur 2.39× vs. 2.10–2.30× longer than broad; chela without pedicel 2.64× vs. 2.30–2.40× longer than broad); from *P.robustus* by smaller body length (e.g. body length (♂) 3.47 mm vs. 3.80 mm, (♀) 2.87–3.65 mm vs. 4.60 mm; palpal femur (♂) 2.39× vs. 2.28× longer than broad, length 0.79 mm vs. 0.91 mm; movable finger length 0.57 mm vs. 0.66 mm) and stouter leg IV (♂) (e.g. femoropatella 2.61× vs. 2.40× longer than deep; tibia 3.17× vs. 3.30× longer than deep) and the number of serrula exterior blades (21 vs. 26); from *P.salomonis* by the trait of eyespots (with two distinct eyespots vs. eyespots wanting), the number of serrula exterior blades (21 vs. 23), slender pedipalps (♂) (e.g. palpal femur 2.39× vs. 2.10× longer than broad, length 0.79 mm vs. 0.69 mm; chela with pedicel 2.83× vs. 2.60× longer than broad); from *P.sinensis* by stouter pedipalps (♂) (e.g. palpal patella 1.76× vs. 1.90× longer than broad; chela with pedicel 2.83× vs. 2.40–2.50× longer than broad) and the arrangement of trichobothria (distance between *est* and *esb* further than that of *ist* and *isb* vs. nearly equal to that of *ist* and *isb*); from *P.sumatranus* by the number of serrula exterior blades (21 vs. 24) and larger body size (♂) (e.g. body length 3.47 mm vs. 2.50 mm; palpal femur 2.39× vs. 2.26× longer than broad, length 0.79 mm vs. 0.61 mm; movable chelal finger length 0.57 mm vs. 0.47 mm); from *P.yunnanensis* sp. nov. by slender chela (♂) (e.g. chela with pedicel 2.83× vs. 2.63–2.64× longer than broad) and relative position of trichobothria *t* and *it* (*t* basal to *it* vs. distal to *it*) (With 1906, With 1907, Beier 1932, Beier 1935a, Beier 1935b, Redikorzev 1938, Beier 1951, Morikawa 1953, Sivaraman 1980, Sivaraman 1981, Mathew and Joseph 2019).

### Paratemnoides yunnanensis

Hou, Zhao & Zhang sp. nov.

46A2666C-C08E-53A8-A070-02DB22C01B94

1107CE3C-305E-47C4-80FE-137B0D8AB97A

#### Materials

- **Type status:** Holotype. **Occurrence:** recordedBy: Yannan Mu; individualCount: 1; sex: male; lifeStage: adult; occurrenceID: B500DA93-8D24-56FD-A8C7-EB4CDFC62690; **Taxon:** scientificName: *Paratemnoidesyunnanensis*; **Location:** country: China; stateProvince: Yunnan; county: Mengla; locality: Menglun Town, Xishuangbanna Tropical Botanical Garden; verbatimElevation: 567 m; verbatimCoordinates: 21°55.416′N, 101°16.097′E; **Event:** year: 2019; month: 8; day: 13; **Record Level:** institutionID: the Museum of Hebei University (MHBU); institutionCode: MHBU-YNML19081301
- **Type status:** Paratype. **Occurrence:** recordedBy: Yannan Mu; individualCount: 5; sex: 5 males; lifeStage: adult; occurrenceID: 586952A9-94BF-5A9F-AB8E-BAF7388053DE; **Taxon:** scientificName: *Paratemnoidesyunnanensis*; **Location:** country: China; stateProvince: Yunnan; county: Mengla; locality: Menglun Town, Xishuangbanna Tropical Botanical Garden; verbatimElevation: 567 m; verbatimCoordinates: 21°55.416′N, 101°16.097′E; **Event:** year: 2019; month: 8; day: 13; **Record Level:** institutionID: the Museum of Hebei University (MHBU); institutionCode: MHBU-YNML19081302–06
- **Type status:** Paratype. **Occurrence:** recordedBy: Yannan Mu; individualCount: 39; sex: 39 females; lifeStage: adult; occurrenceID: B6BE62E0-DA43-59B5-916C-48EBF6FC8D6D; **Taxon:** scientificName: *Paratemnoidesyunnanensis*; **Location:** country: China; stateProvince: Yunnan; county: Mengla; locality: Menglun Town, Xishuangbanna Tropical Botanical Garden; verbatimElevation: 567 m; verbatimCoordinates: 21°55.416′N, 101°16.097′E; **Event:** year: 2019; month: 8; day: 13; **Record Level:** institutionID: the Museum of Hebei University (MHBU); institutionCode: MHBU-YNML19081307–45

#### Description

**Figure 13.**
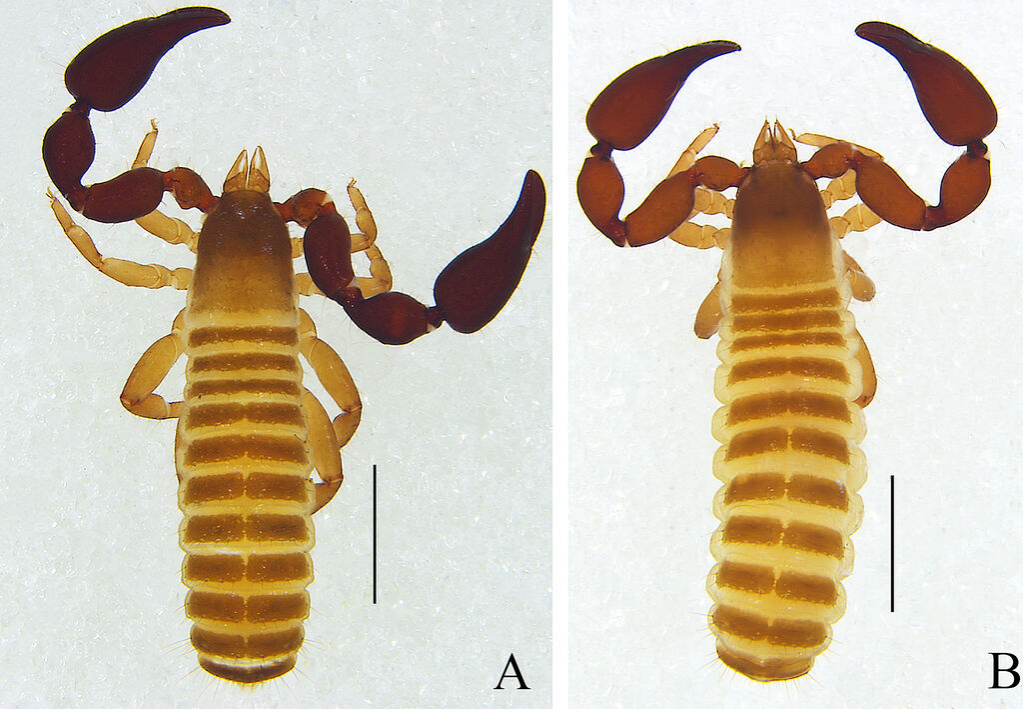
*Paratemnoidesyunnanensis* sp. nov. **A** holotype male, habitus (dorsal view); **B** paratype female, habitus (dorsal view). Scale bars: 1.00 mm.

**Figure 14.**
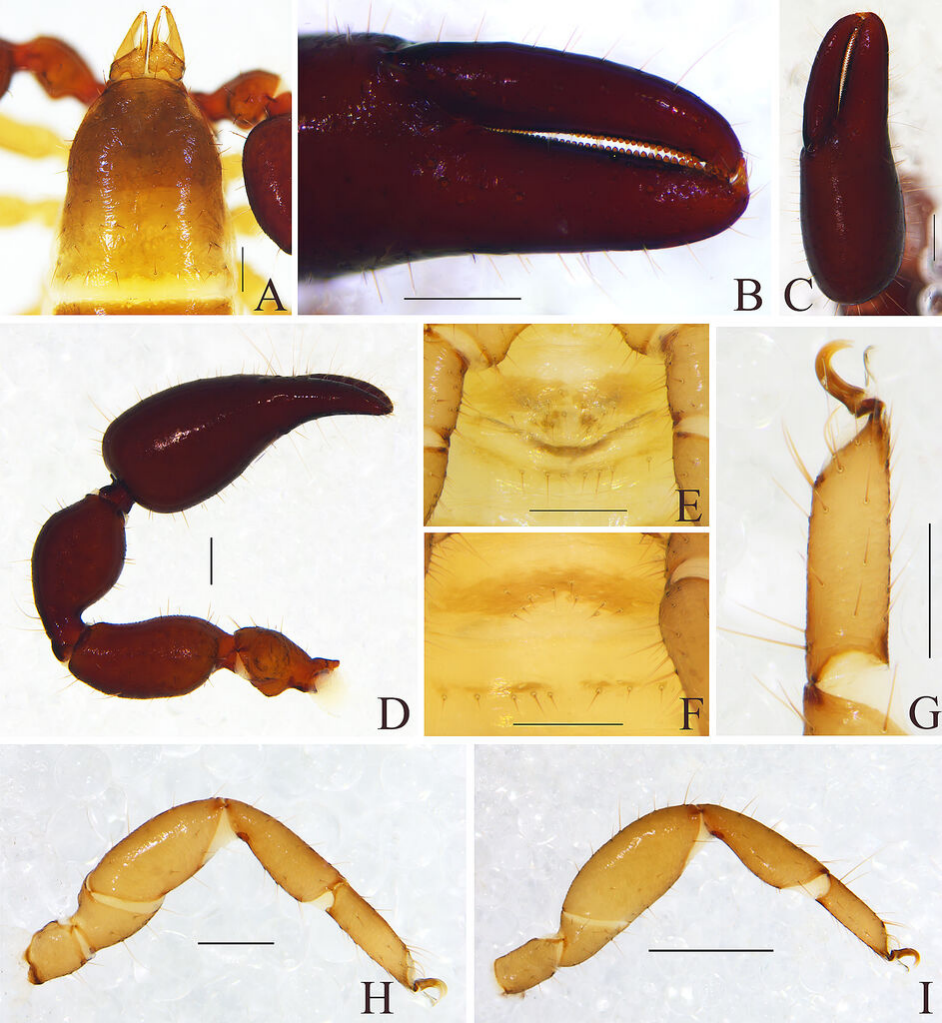
*Paratemnoidesyunnanensis* sp. nov., holotype male (**A–E, G–I**), paratype female (**F**) **A** carapace (dorsal view); **B** left chelal fingers (lateral view); **C** left chela (lateral view); **D** left pedipalp (dorsal view); **E** male genital area (ventral view); **F** female genital area (ventral view); **G** tarsus of left leg IV (lateral view); **H** left leg I (lateral view); **I** left leg IV (lateral view). Scale bars: 0.50 mm (**I**); 0.20 mm (**A–H**).

**Figure 15.**
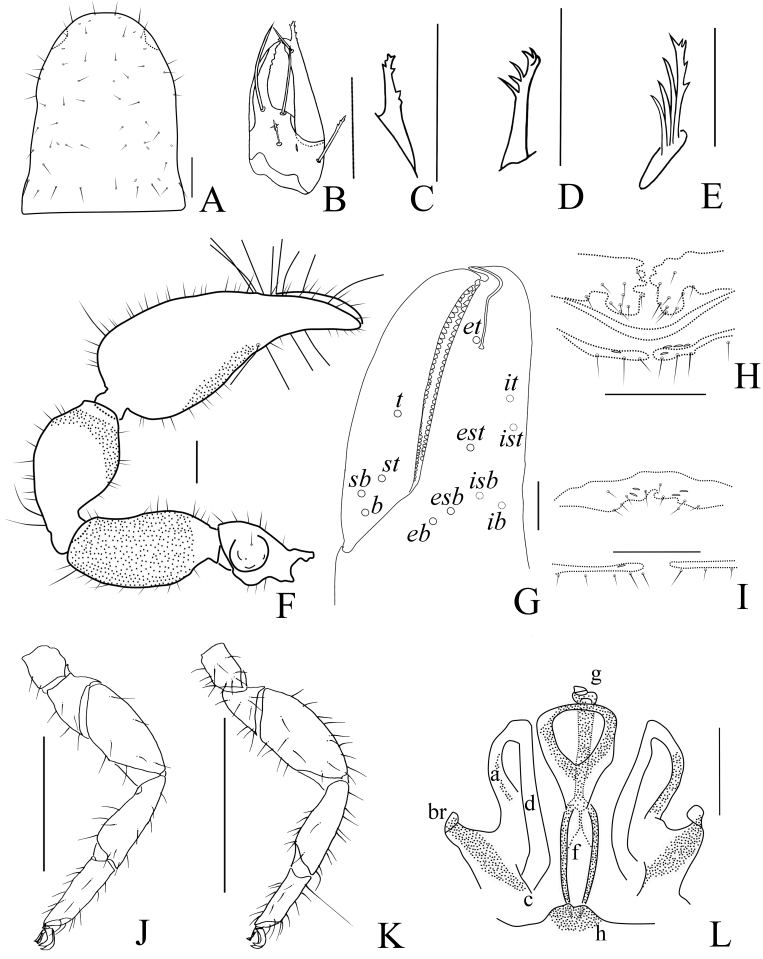
*Paratemnoidesyunnanensis* sp. nov., holotype male (**A–C, E–H, J–L**), paratype female (**D, I**) **A** carapace (dorsal view); **B** left chelicera (dorsal view); **C** male galea; **D** female galea; **E** rallum; **F** left pedipalp (dorsal view); **G** left chelal fingers (lateral view), with details of trichobothrial pattern; **H** male genital area (ventral view); **I** female genital area (ventral view); **J** left leg I (lateral view); **K** left leg IV (lateral view); **L** male genital organ. Scale bars: 1 mm (**K**); 0.50 mm (**J**); 0.25 mm (**D**); 0.20 mm (**A, H, I**); 0.10 mm (**B–G, L**).

Males (holotype and paratypes) (Fig. 13A, Fig. 14A–E, G–I, Fig. 15A–C, E–H and J–L). Colour: anterior half of carapace brown, but paler in posterior half; pedipalps reddish-brown; tergites yellowish-brown; remainder light yellow.

Carapace (Figs 14, 15A): 1.05–1.13× longer than broad; surface smooth, without furrow; anterior half slightly darker than posterior half; with two distinct eyespots situated near anterior margin of carapace; anterior margin with four setae, posterior margin with eight or nine setae, 60–61 in total, each seta acicular and very slightly curved.

Chelicera (Fig. 15B–C and E): much smaller than carapace length; surface smooth; four setae (*sbs* absent; *bs* shorter than others) and two lyrifissures (exterior condylar lyrifissure and exterior lyrifissure) present on hand; movable finger with one slightly curved galeal seta; *bs* and *es* dentate apically, *is* and *ls* long and acute. Fixed finger with four large retrorse teeth and three small apical teeth, movable finger with a long broadly dentated subapical lobe and two small sub-terminal teeth; galea present, slender and with five small terminal dentations and two small lateral dentations (Fig. 15C). Serrula interior connected to fixed finger for entire length, proximally modified to form velum, serrula exterior with 23–25 blades, the basal one longest; lamina exterior present. Rallum composed of four blades, the basal two blades shorter than others, the distal one dentated anteriorly, remainder smooth (Fig. 15E).

Pedipalp (Fig. 14B–D and Fig. 15F–G): stout, trochanter 1.52–1.56×, femur 2.17–2.41×, patella 1.77–1.87×, chela with pedicel (without pedicel) 2.63–2.64× (2.50–2.51×), hand without pedicel 1.44–1.49× longer than broad; movable chelal finger 0.75× longer than hand without pedicel. Setae generally long and acuminate. Prolateral surface of femur, patella and hand granular; trochanter with two well-developed conical tubercles. Fixed chelal finger with eight trichobothria, movable chelal finger with four trichobothria: *eb* and *esb* situated at base of fixed finger on retrolateral face, *esb* slightly distal to *eb*; *ib* and *isb* situated at base of fixed finger on prolateral face, *isb* slightly distal to *ib*; *est* in the middle of fixed finger; *et* near sub-distal of fixed finger; *est* closer to *esb* than to *et*; *it* distal to *est* and proximal to *et*; *ist* slightly distal to *est* and proximal to *it*; *it* closer to *ist* than to fingertip; distance between *est* and *esb* nearly equal to that of *ist* and *isb*; distance between *it* and fingertip further than distance between *ist* and *isb*; *b* and *sb* situated at base of movable finger on retrolateral face; *t* in the middle of movable finger and at same level as *ist*; *sb* slightly closer to *b* than to *st*; *st* closer to *sb* than to *t* (Fig. 15G). Venom apparatus only present in fixed chelal finger, venom ducts curved and short, terminating in inflated nodus ramosus between *et* and *est*, very close to *et.* Both chelal fingers with a row of acute teeth, spaced contiguously along the margin, slightly rounded proximally: fixed chelal finger with 33–34 teeth; movable chelal finger with 46–48 teeth (nearly as large as teeth on fixed chelal finger); without accessory teeth (Fig. 15G). Femur without long tactile setae. Movable chelal finger slightly curved in lateral view (Fig. 14B and Fig. 15G).

Opisthosoma: generally typical, all setae long, acuminate and biseriate; pleural membrane longitudinally striate, without setae. Tergites I and XI undivided and others incompletely divided, tergal chaetotaxy I–XI: 10: 5–6: 5–6: 6–7: 7–9: 6–8: 7–8: 6–8: 7–8: 6–7+ (4T): 13 + (2T). All sternites (except sternite XI) divided, sternal chaetotaxy IV–XI: 5–5: 7–8: 8–9: 7–9: 8–9: 7–9: 6–7 + (4T): 10 + (4T). Anus (tergite Ⅻ and sternite Ⅻ) without raised rim. Anterior genital operculum with five or seven setae on each side, posterior margin with five setae. Male genitalia (Fig. 14E, Fig. 15H and L) well-developed: lateral apodemes (a) relatively small; the hooked branch (br) well-developed, bowed distally and terminating in a plate-like tip; the proximal part with a nearly pale sclerotised bar (c), distinctly curved; the longitudinal fold of medial diverticula (d) vestigial; the ejaculatory canal atrium (e) not well-developed, curved distally; the lateral rods (f) short and diverging proximally; the tip of dorsal apodeme (g) completely joined; the ventral diverticulum (h) bilobed; genital atrium without genital setae.

Legs (Fig. 14G–I, Fig. 15J and K): generally typical, fairly smooth, slightly stout; junction between femora and patellae I and II oblique. Femoropatella of leg Ⅳ 2.72–2.74× longer than deep; tibia 3.00–3.16× longer than deep; with basal tactile setae on tarsal segment: tarsus 3.15–3.17× longer than deep (TS = 0.15–0.16); subterminal tarsal setae arcuate and acute. Arolium slightly shorter than claws, not divided; claws smooth.

Adult females (Fig. 13B, Fig. 14F and Fig. 15I): Mostly same as the males, but a little larger and paler. Chelicera: hand with four setae; galea with six branchlets; serrula exterior with 21–23 blades, the basal one longest. Pedipalps: stout, trochanter 1.52–1.65, femur 2.33, patella 1.88–1.92, chela (with pedicel) 2.72–2.79, chela (without pedicel) 2.55–2.65, hand (without pedicel) 1.53–1.56× longer than broad, movable chelal finger 0.70–0.71× longer than hand without pedicel; fixed chelal finger with 35 teeth; movable chelal finger with 48 teeth. Opisthosoma: tergites I–III and XI undivided and others incompletely divided, tergal chaetotaxy I–XI: 12: 11: 11: 6–7:7–8: 7–7: 7–9: 7–9: 7–9: 6–7 (4T): 13 (2T). All sternites divided, sternal chaetotaxy IV–XI: 4–6: 9–8: 8–10: 7–8: 8–9: 7–9: 6–7 (4T): 11 (4T). Female genitalia: simple, spermathecae provided with separated median cribriform plates; anterior genital operculum with ten setae on each side, posterior margin with eight setae. Legs: femoropatella of leg Ⅳ 2.65–2.77× longer than deep; tibia 3.00–3.05× longer than deep; with basal tactile setae on tarsal segment: tarsus 3.07–3.42× longer than deep.

Dimensions (length/breadth or, in the case of the legs, length/depth in mm; ratios in parentheses). Males (females in parentheses): body length 3.61–3.78 (3.94–4.42). Carapace 0.85–0.89/0.79–0.81 (0.92/0.78–0.79). Pedipalp: trochanter 0.41–0.42/0.27 (0.43–0.44/0.26–0.29), femur 0.76–0.82/0.34–0.35 (0.77–0.84/0.33–0.36), patella 0.69–0.71/0.38–0.39 (0.69–0.75/0.36–0.40), chela (with pedicel) 1.32–1.34/0.50–0.51 (1.34–1.44/0.48–0.53), chela (without pedicel) 1.25–1.28 (1.27–1.35), hand (without pedicel) 0.72–0.76 (0.75–0.81), movable finger length 0.54–0.57 (0.53–0.57). Leg I: trochanter 0.15/0.14 (0.15–0.17/0.14–0.16), femur 0.26/0.17–0.20 (0.25–0.27/0.19–0.22), patella 0.42/0.19 (0.41–0.46/0.18–0.20), tibia 0.38/0.13 (0.38–0.41/0.13–0.14), tarsus 0.33/0.10 (0.32–0.34/0.09–0.10). Leg IV: trochanter 0.30–0.32/0.18–0.19 (0.31–0.34/0.15–0.18), femoropatella 0.85–0.87/0.31–0.32 (0.86–0.90/0.31–0.34), tibia 0.60/0.19–0.20 (0.58–0.63/0.19–0.21), tarsus 0.38–0.41/0.12–0.13 (0.41–0.43/0.12–0.14).

#### Diagnosis

This new species is characterised by (see taxon discussion for more details): carapace with two distinct eyespots and 60–61 setae; anterior half of carapace darker than posterior half; palpal femur 2.17–2.41 (♂), 2.33 (♀), chela with pedicel 2.63–2.64 (♂), 2.72–2.79 (♀) × longer than broad, chela without pedicel 2.50–2.51 (♂), 2.55–2.65 (♀) × longer than broad; prolateral surface of femur, patella and hand granular; movable chelal finger with 46–48 teeth.

#### Etymology

Named after the type locality, Yunnan (China).

#### Distribution

**Figure 16.**
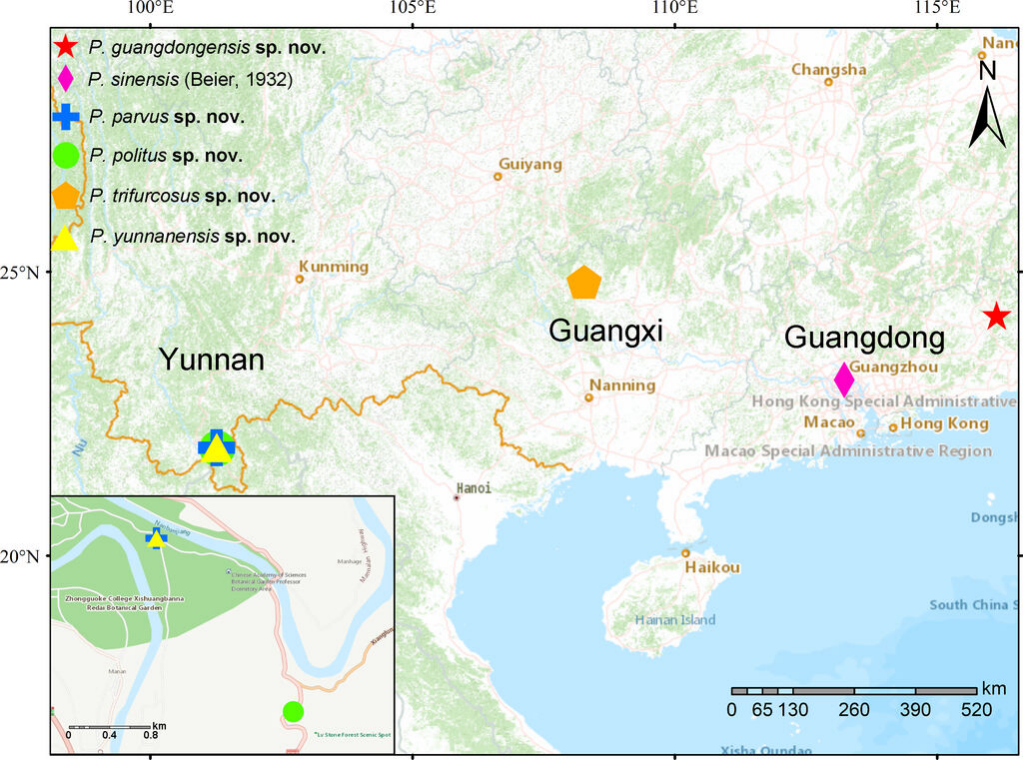
Type localities of *Paratemnoides* species in China.

China (Yunnan) (Fig. 16).

#### Taxon discussion

*Paratemnoidesyunnanensis* sp. nov. is similar to *P.sinensis*, but differs by the presence of more movable chelal finger teeth (46–48 vs. 43) and slightly slender chela (♂) (chela with pedicel 2.63–2.64× vs. 2.40–2.50× longer than broad).

*Paratemnoidesyunnanensis* sp. nov. can be distinguished from *P.assimilis* by the number of posterior margin setae on the carapace (8–9 vs. 6), the number of serrula exterior blades (23–25 vs. 21), larger body length and stouter pedipalps (e.g. body length (♀) 3.94–4.42 mm vs. max. 3.70 mm; palpal patella (♂) 1.77–1.87× vs. 2.00× longer than broad, length 0.69–0.71 mm vs. 0.77 mm); from *P.borneoensis* by larger body size and slender pedipalps (♀) (e.g. body length 3.94–4.42 mm vs. 3.50 mm; palpal femur length 0.77–0.84 mm vs. 0.68 mm; chela with pedicel 2.72–2.79× vs. 2.60× longer than broad); from *P.curtulus* by the arrangement of trichobothria (e.g. distance between *est* and *esb* nearly equal to that of *ist* and *isb* vs. shorter to that of *ist* and *isb*) and slender chela (♂) (chela with pedicel 2.63–2.64× vs. 2.19× longer than broad, length 1.32–1.34 mm vs. 1.05 mm); from *P.guangdongensis* sp. nov. by stouter chela (♂) (e.g. chela with pedicel 2.63–2.64× vs. 2.73× longer than broad); from *P.indicus* by the presence of more setae on the carapace (60–61 vs. 46), more movable chelal finger teeth (46–48 vs. 42) and larger body size (♀) (e.g. body length 3.94–4.42 mm vs. 3.50 mm; palpal femur 2.33× vs. 2.00× longer than broad, length 0.77–0.84 mm vs. 0.72 mm); from *P.japonicus* by the trait of eyes (with two distinct eyespots vs. eyespots absent), the number of serrula exterior blades (23–25 vs. 18) and larger body size (♂) (e.g. body length 3.61–3.78 mm vs. 2.97 mm); from *P.laosanus* by slender chela and legs (♂) (e.g. chela without pedicel 2.50–2.51× vs. 2.20–2.30× longer than broad; femoropatella of leg IV 2.72–2.74× vs. 2.60× longer than deep; tibia of leg IV 3.00–3.16× vs. 2.80× longer than deep); from *P.mahnerti* by the presence of more setae on the carapace (60–61 vs. 40), the number of serrula exterior blades (23–25 vs. 19) and relative position of trichobothrium *st* (*st* situated closer to *sb* than to *t* vs. midway between *sb* and *t*); from *P.pallidus* by slender pedipalps (♀) (e.g. chela with pedicel 2.72–2.79× vs. 2.20× longer than broad; palpal femur 2.33× vs. 2.20× longer than broad); from *P.parvus* sp. nov. by more movable chelal finger teeth (♂) (46–48 vs. 41–42) and relative position of trichobothria *ist* and *est* (i.e. *ist* situated distal to *est* vs. basal to *est*); from *P.philippinus* by larger body size (♂) (e.g. body length 3.61–3.78 mm vs. 3.50 mm; chela with pedicel 2.63–2.64× vs. 2.20× longer than broad); from *P.plebejus* by stouter pedipalps (e.g. palpal femur (♂) 2.17–2.41×, (♀) 2.33× vs. (♂) 2.11×, (♀) 2.00× longer than broad) and the trait of tergites (♀) (e.g. tergite I undivided in the new species while divided in the latter); from *P.pococki* by darker body colour (carapace and tergites brown vs. pale brown) and larger body size and slender pedipalps (♀) (body length 3.94–4.42 mm vs. 3.87 mm; palpal femur 2.33× vs. 2.20× longer than broad, length 0.77–0.84 mm vs. 0.62 mm; movable chelal finger length 0.53–0.57 mm vs. 0.46 mm); from *P.politus* sp. nov. by slender pedipalps (♂) (e.g. palpal femur 2.17–2.41× vs. 1.94–2.00× longer than broad) and relative position of trichobothria *t* and *it* (*t* basal to *it* vs. distal to *it*) ; from *P.redikorzevi* by the number of serrula exterior blades (23–25 vs. 20), larger body size and slender pedipalps (e.g. body length (♂) 3.61–3.78 mm vs. 3.00 mm; palpal femur (♀) 2.33× vs. 2.18–2.19× longer than broad, length 0.77–0.84 mm vs. 0.70–0.72 mm); from *P.robustus* by stouter pedipalps and legs (♂) (e.g. chela with pedicel 2.63–2.64× vs. 2.20× longer than broad; femoropatella of leg IV 2.72–2.74× vs. 2.40× longer than deep; tibia of leg IV 3.00–3.16× vs. 3.30× longer than deep); from *P.salomonis* by the trait of eyespots (with two distinct eyespots vs. eyespots wanting), larger body length and the slender pedipalps (♂) (e.g. body length 3.61–3.78 mm vs. 3.50 mm; palpal femur 2.17–2.41× vs. 2.10× longer than broad, length 0.76–0.82 mm vs. 0.69 mm; chela without pedicel 2.50–2.51× vs. 2.40× longer than broad); from *P.sumatranus* by larger body size and slender pedipalps (e.g. body length (♂) 3.61–3.78 mm vs. 2.50 mm, (♀) 3.94–4.42 mm vs. 2.80–3.30 mm; chela with pedicel (♂) 2.63–2.64× vs. 2.80–2.90× longer than broad, movable chelal finger length 0.54–0.57 mm vs. 0.47 mm) and stouter leg IV (♂) (e.g. femoropatella 2.72–2.74× vs. 3.00×, tibia 3.00–3.16× vs. 3.60×, tarsus 3.15–3.17× vs. 4.50× longer than deep); from *P.trisulcus* sp. nov. by stouter chela (♂) (e.g. chela with pedicel 2.63–2.64× vs. 2.83× longer than broad) and relative position of trichobothria *t* and *it* (*t* distal to *it* vs. basal to *it*) (With 1906, With 1907, Beier 1932, Beier 1935a, Beier 1935b, Redikorzev 1938, Beier 1951, Morikawa 1953, Sivaraman 1980, Sivaraman 1981, Mathew and Joseph 2019).

## Identification Keys

### Key to the species of *Paratemnoides* from China

**Table:** 

1	Three rallum blades anteriorly dentated (♀); male genitalia: the distal part (l) of lateral apodemes well-developed	***P.trisulcus* sp. nov.**
–	Only the distal rallum blade anteriorly dentated (♀); male genitalia: the distal part (l) of lateral apodemes vestigial	2
2	Only surface of palpal femur granular	***P.politus* sp. nov.**
–	Surface of multiple palpal segments granular	3
3	Movable chelal finger with 54–56 teeth	***P.guangdongensis* sp. nov.**
–	Movable chelal finger with less than 48 teeth	4
4	Distance between *est* and *esb* further than that of *ist* and *isb*	***P.parvus* sp. nov.**
–	Distance between *est* and *esb* nearly equal to that of *ist* and *isb*	5
5	Movable chelal finger with 46–48 teeth; chela with pedicel (♂) 2.63–2.64× longer than broad	***P.yunnanensis* sp. nov.**
–	Movable chelal finger with 43 teeth; chela with pedicel (♂) 2.40–2.50× longer than broad	***P.sinensis* (Beier, 1932)**

## Supplementary Material

XML Treatment for Paratemnoides guangdongensis

XML Treatment for Paratemnoides parvus

XML Treatment for Paratemnoides politus

XML Treatment for Paratemnoides trisulcus

XML Treatment for Paratemnoides yunnanensis
